# Direct transposition of native DNA for sensitive multimodal single-molecule sequencing

**DOI:** 10.1038/s41588-024-01748-0

**Published:** 2024-05-09

**Authors:** Arjun S. Nanda, Ke Wu, Iryna Irkliyenko, Brian Woo, Megan S. Ostrowski, Andrew S. Clugston, Leanne C. Sayles, Lingru Xu, Ansuman T. Satpathy, Hao G. Nguyen, E. Alejandro Sweet-Cordero, Hani Goodarzi, Sivakanthan Kasinathan, Vijay Ramani

**Affiliations:** 1 Gladstone Institute for Data Science and Biotechnology, Gladstone Institutes, San Francisco, CA USA; 2 Department of Biochemistry and Biophysics, University of California, San Francisco, San Francisco, CA USA; 3 Helen-Diller Cancer Center, San Francisco, CA USA; 4 Department of Pediatrics, University of California, San Francisco, San Francisco, CA USA; 5 Department of Pathology, Stanford University, Stanford, CA USA; 6 Parker Institute for Cancer Immunotherapy, San Francisco, CA USA; 7 Gladstone-University of California, San Francisco Institute for Genomic Immunology, Gladstone Institutes, San Francisco, CA USA; 8 Bakar Computational Health Sciences Institute, San Francisco, CA USA; 9 Division of Rheumatology, Department of Pediatrics, Stanford University, Stanford, CA USA

**Keywords:** Sequencing, Epigenetics, Epigenomics

## Abstract

Concurrent readout of sequence and base modifications from long unamplified DNA templates by Pacific Biosciences of California (PacBio) single-molecule sequencing requires large amounts of input material. Here we adapt Tn5 transposition to introduce hairpin oligonucleotides and fragment (tagment) limiting quantities of DNA for generating PacBio-compatible circular molecules. We developed two methods that implement tagmentation and use 90–99% less input than current protocols: (1) single-molecule real-time sequencing by tagmentation (SMRT-Tag), which allows detection of genetic variation and CpG methylation; and (2) single-molecule adenine-methylated oligonucleosome sequencing assay by tagmentation (SAMOSA-Tag), which uses exogenous adenine methylation to add a third channel for probing chromatin accessibility. SMRT-Tag of 40 ng or more human DNA (approximately 7,000 cell equivalents) yielded data comparable to gold standard whole-genome and bisulfite sequencing. SAMOSA-Tag of 30,000–50,000 nuclei resolved single-fiber chromatin structure, CTCF binding and DNA methylation in patient-derived prostate cancer xenografts and uncovered metastasis-associated global epigenome disorganization. Tagmentation thus promises to enable sensitive, scalable and multimodal single-molecule genomics for diverse basic and clinical applications.

## Abstract (web summary)

Two low-input tagmentation-based long-read sequencing methods, single-molecule real-time sequencing by tagmentation (SMRT-Tag), which identifies genetic variation and CpG methylation, and single-molecule adenine-methylated oligonucleosome sequencing assay by tagmentation (SAMOSA-Tag), which detects chromatin accessibility, are presented. Application of SAMOSA-Tag to prostate cancer patient-derived xenograft samples identifies metastasis-associated epigenomic alterations.

## Main

Third-generation single-molecule sequencing (SMS) technologies deliver accurate, multimodal readouts of genetic sequence and nucleobase modifications on kilobase (kb)-length to megabase-length nucleic acid templates^1^. SMS has facilitated the characterization of previously intractable structural variants and repetitive regions^2,3^, assembly of gapless human genomes and high-resolution functional genomics of DNA^4–8^ and RNA^9,10^. The intrinsic multimodality of SMS has been exploited by chromatin profiling methods, such as the single-molecule adenine-methylated oligonucleosome sequencing assay (SAMOSA)^4,11^, Fiber-seq^5^, Nanopore sequencing of nucleosome occupancy and methylome^7^ and others^6,8,12^. These approaches establish a paradigm for encoding functional genomic information (for example, histone–DNA and transcription factor–DNA interactions) as separate SMS ‘channels’ concurrently with primary sequence and endogenous epigenetic marks, such as CpG methylation.

Over the past decade, improvements in cost, data quality, read length and computational tools have driven rapid maturation of the Pacific Biosciences of California (PacBio) and Oxford Nanopore Technologies (ONT) SMS platforms. For example, the cost of PacBio sequencing has decreased from US$2,000 to US$35 per gigabase (Gb), concomitant with increases in yield (100 Mb to 90 Gb per instrument run), read length (from approximately 1.5 kb to 15–20 kb) and accuracy (from approximately 85% to more than 99.95%)^13^. A key limitation of PacBio SMS is the amount of input DNA required for PCR-free library preparation (typically at least 1–5 µg, or 150,000–750,000 human cells; Supplementary Note 1) owing to sample losses during mechanical or enzymatic fragmentation, adapter ligation and serial reaction cleanups. While low-input protocols are available, they typically rely on PCR amplification, which erases modified bases and may introduce biases. This obstacle has limited the primary use of SMS to genome assembly and medical genetics, precluding analyses of rare clinical samples and post-mitotic cell populations, single cells and microorganisms.

Simultaneous transposition of sequencing adapters and template DNA fragmentation (that is, tagmentation) using hyperactive Tn5 transposase poses an attractive solution to this problem^14^. The reduced input requirement and workflow complexity of Tn5-based short-read library preparation has transformed bulk genome, epigenome and transcriptome profiling^15–17^ and enabled single-cell and spatial monoplex^18–20^ and multiomic sequencing^21–23^. Reasoning that the high efficiency of tagmentation and consolidation of protocol steps would similarly facilitate low-input SMS, we optimized transposition of hairpin adapters to yield long circular molecules for PacBio sequencing^24^. We then applied this principle to develop two PCR-free multimodal methods: (1) single-molecule real-time sequencing by tagmentation (SMRT-Tag) for assaying the genome and epigenome; and (2) SAMOSA by tagmentation (SAMOSA-Tag), which adds a concurrent channel for mapping chromatin structure. SMRT-Tag accurately detected genetic and epigenetic variants from as little as 40 ng of DNA. SAMOSA-Tag maps of single-fiber CTCF and nucleosome occupancy and CpG methylation uncovered metastasis-associated global chromatin deregulation in technically challenging patient-derived xenografts (PDXs) from a patient with prostate cancer. These results extend tagmentation to PacBio library preparation and have the potential to enable sensitive, scalable and cellularly resolved single-molecule genomics.

## Results

### Tn5 transposition produces PacBio-compatible molecules

**Fig. 1:**
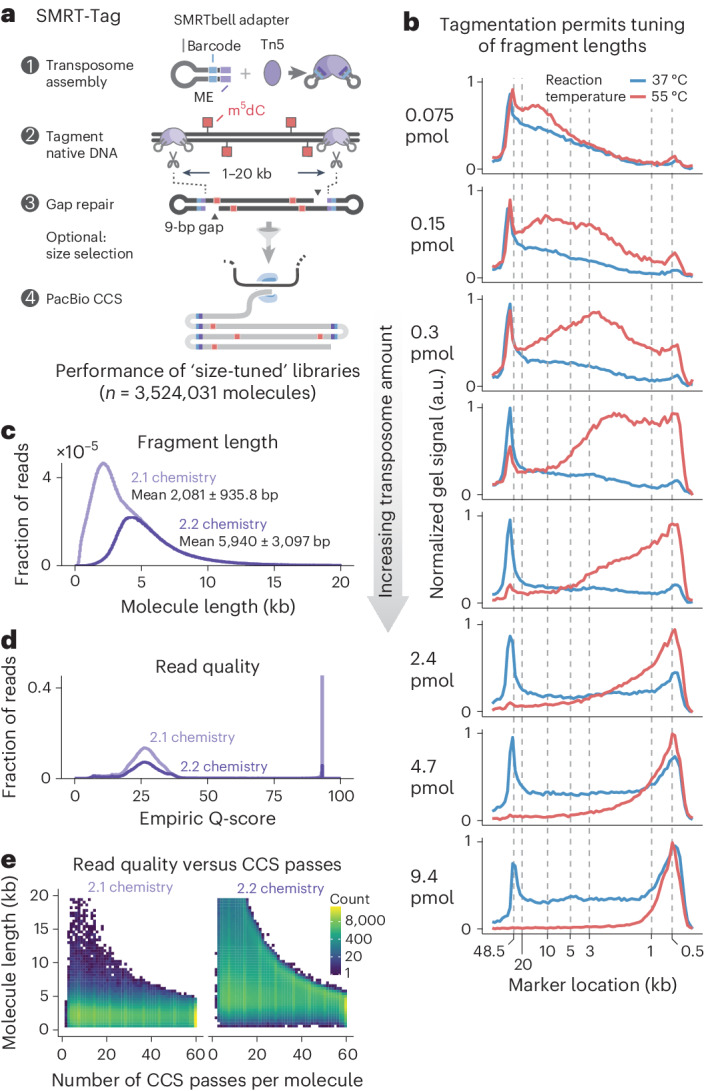
Tagmentation enables tunable and sensitive single-molecule real-time sequencing. **a**, In SMRT-Tag, hairpin adapter-loaded Tn5 transposase was used to fragment DNA into kb-scale fragments. The 9-nt gaps introduced by transposition were closed via optimized gap repair, while exonuclease digestion enriched for the covalently closed templates required for PacBio sequencing. **b**, Varying concentration of hairpin-loaded transposomes and reaction temperature tuned fragmentation of gDNA over a size range of 2–10 kb. **c**, PacBio CCS fragment lengths for SMRT-Tag libraries fractionated into short and long molecules optimal for PacBio polymerases 2.1 (light purple) and 2.2 (dark purple) chemistries, respectively. The distribution for the long-fragment library (2.2 chemistry) has a tail that extends beyond 20 kb. **d**, Empiric Q-score distributions for the 2.1 and 2.2 libraries. **e**, Heatmap of logarithmically scaled counts of CCS length as a function of the number of CCS passes per molecule. Source data

Two technical factors need to be addressed to efficiently generate long (>1 kb) molecules for PacBio SMS via transposition of hairpin adapters into genomic DNA (gDNA) (illustrated with the SMRT-Tag workflow; Fig. 1a). First, the conventional Tn5 enzyme used in many short-read sequencing methods optimally produces 100–500 bp fragments. Therefore, we selected a triple-mutant Tn5 enzyme (hereafter referred to as Tn5), which permits concentration-dependent control of fragment size^25^. We loaded Tn5 with custom oligonucleotides consisting of the hairpin PacBio adapter and mosaic end sequences needed to assemble transposomes. Analytical electrophoresis of gDNA tagmented with adapter-loaded Tn5 at varying reaction conditions confirmed generation of fragments more than 1-kb long, which are favored at low transposome concentrations and temperature (Fig. 1b). Additional considerations for controlling library size are detailed below and in Supplementary Note 2.

Second, Tn5 transposition introduces 9-nt gaps into template molecules^26^ (Fig. 1a), which must be sealed for productive SMS. While hairpin transposition has been reported for short-read, single-cell genomics^18^, and Tn5 is used in some ONT protocols, efficient gap repair to create closed, circular molecules has, to our knowledge, not been reported. We thus tested 62 conditions (Supplementary Table 1) to optimize gap filling. Two enzyme combinations proved to be the most robust based on yield (Supplementary Fig. 1) and electrophoretic fragment lengths (Supplementary Fig. 2) of gDNA subjected to tagmentation, repair and exonuclease digestion to select for closed circles: Phusion polymerase and Taq DNA ligase (‘Phusion/Taq’) and T4 DNA polymerase and Ampligase (‘T4/Ampligase’). These produced exonuclease-resistant libraries from as little as 50 ng gDNA, with typical yields of more than 20% of input mass (Supplementary Table 2). In all subsequent experiments, we used Phusion/Taq because it provided significantly higher yields on gDNA than T4/Ampligase (*P* = 0.0093, two-sided *t*-test).

### SMRT-Tag produces tunable libraries for multiplexed SMS

We applied direct transposition in SMRT-Tag, a simple method for whole-genome analysis, and explored library and sequencing characteristics. To evaluate the sequencing efficiency of SMRT-Tag, we tagmented 120 ng of HG002 gDNA (equivalent to approximately 20,000 human cells) in eight separate reactions and used solid-phase reversible immobilization (SPRI) beads to fractionate the resulting libraries for sequencing using PacBio’s proprietary 2.1 and 2.2 polymerases optimized for short and long templates, respectively (Supplementary Note 2). Circular consensus sequencing (CCS) read length distributions of the 3,524,301 molecules (14.3 Gb total) sequenced over two runs were concordant with size selection and polymerase choice (Fig. 1c; 2,081 ± 935.8 bp versus 5,940 ± 3,097 bp for polymerases 2.1 and 2.2, respectively; mean ± s.d.). The per-read quality scores (Q-scores; Fig. 1d) and number of CCS passes (Fig. 1e) were sufficient for PacBio high-fidelity (‘HiFi’) sequencing with more than 99% (>Q20) base accuracy, which typically requires 5 or more redundant passes per molecule.

To assess demultiplexing using the 8-nt barcode included in the SMRT-Tag hairpin adapter (Fig. 1a), we first performed low-pass sequencing of libraries pooled after tagmentation, gap repair and exonuclease digestion of gDNA from the extensively genotyped HG002, HG003 and HG004 human trio (in total, seven 80-ng reactions sequenced to 0.75× HG002, 1.39× HG003 and 1.30× HG004 depths; Supplementary Fig. 3a). We inspected the left and right barcodes of molecules, which were identical (99.9% concordance; Supplementary Fig. 3b). Taking advantage of the pedigree to query genotype mixing of multiplexed libraries, we confirmed that HG003 and HG004 (unrelated parents) shared few private single-nucleotide variants (SNVs) (0.60% HG003 versus HG004; 0.67% HG004 versus HG003), while HG002 (child) was a mixture of parental genotypes (33.1% overlap; Supplementary Fig. 3c). Second, to determine if samples could be multiplexed immediately after tagmentation, we sequenced gDNA libraries from four separate reactions pooled before gap repair and exonuclease digestion (Supplementary Fig. 3d). Barcode concordance (99.9%; Supplementary Fig. 3e) and Smith–Waterman barcode alignment scores reported by the lima demultiplexer (mean = 97.9, s.d. = 6.78, normalized scale 0–100; Supplementary Fig. 3f) were excellent. This confirmed that there was no tagging of previously transposed molecules during gap repair, exonuclease cleanup and pooling, and was consistent with the zero turnover activity of Tn5.

Finally, to illustrate the tunability of SMRT-Tag, we tagmented gDNA at varying Tn5 concentrations and reaction temperatures, and multiplexed libraries for sequencing. The resulting read length distributions confirmed that the Tn5:DNA ratio and temperature can be varied to shift library size distributions (Supplementary Fig. 4). The mean and s.d. of fragment lengths were respectively controllable over nearly 11-fold and 18-fold dynamic ranges, offering an important reference point for implementing the approach (Supplementary Fig. 4c). For all experiments, unless otherwise noted, libraries were multiplexed to minimize sequencing cost. Supplementary Note 1 details the rationale for this, and the design choices for library preparation, polymerase binding and flow cell loading. Sequencing and quality metrics for all libraries and pooling strategies for analyses are shown in Supplementary Tables 3 and 4. We conclude that SMRT-Tag generates multiplexable PCR-free PacBio libraries from low-input DNA amounts for multiplex sequencing.

### SMRT-Tag accurately detects genetic and epigenetic variation

**Fig. 2:**
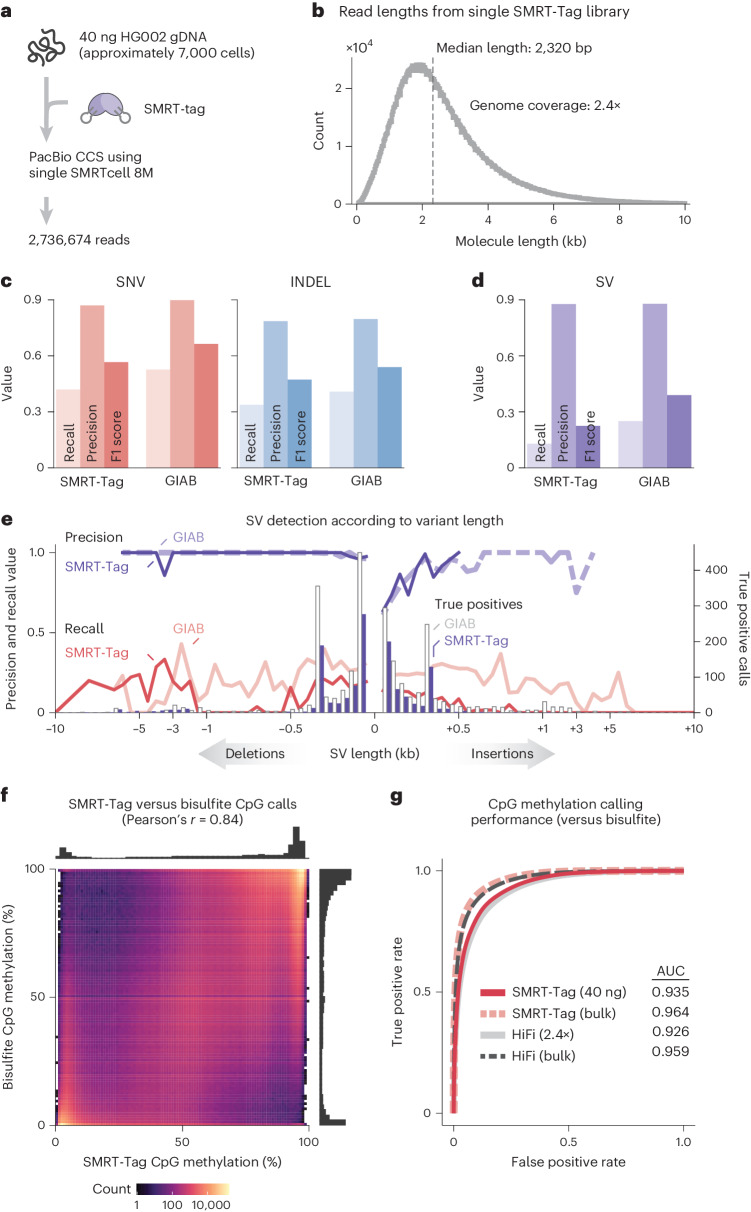
SMRT-Tag enables accurate genotyping and epigenotyping of low-input samples. **a**, To establish whether low-input SMRT-Tag libraries could be sequenced to sufficient depth, we tagmented 40 ng gDNA (equivalent to approximately 7,000 human cells) from GIAB reference individual HG002 and sequenced the resulting library on a single flow cell. **b**, Read length distribution of the 40-ng SMRT-Tag library. **c**,**d**, Precision, recall and F1 scores for DeepVariant SNV and indel calls (**c**) and pbsv SV calls (**d**) from 40-ng SMRT-Tag and coverage-matched ligation-based PacBio data compared with GIAB HG002 variant calling benchmarks. **e**, Precision, recall and number of true positive calls for SVs binned according to size for 40-ng SMRT-Tag and coverage-matched ligation-based data benchmarked against GIAB HG002 SV calls. **f**, Comparison of SMRT-Tag primrose and HG002 bisulfite CpG methylation. **g**, AUCs for CpG methylation detected using 40-ng SMRT-Tag, pooled SMRT-Tag (not coverage-matched) and ligation-based PacBio compared with bisulfite sequencing.

We next sought to establish the sensitivity and variant calling accuracy of SMRT-Tag. We first determined whether libraries could be generated at the minimum on-plate loading concentration (OPLC) for PacBio Sequel II flow cells of 20–40 pM. We sequenced one SMRT-Tag library generated from 40 ng HG002 gDNA (approximately 7,000 human cell equivalents) achieving 37 pM OPLC (Fig. 2a and Supplementary Note 1). A single flow cell yielded 2,736,674 CCS reads with 2.32-kb median length, equivalent to approximately 2.43× genome coverage (Fig. 2b). While this depth is suboptimal for routine genotyping applications, we next asked whether data quality was sufficient for variant detection. We called SNVs and insertion-deletion (indel) variants using DeepVariant and structural variants (SVs) with pbsv from low-input SMRT-Tag and coverage-matched ligation-based libraries sequenced by the Genome in a Bottle (GIAB) consortium^27^. To evaluate accuracy, we benchmarked detected variants against the gold standard GIAB high-confidence HG002 callset^28^ (Fig. 2c–e). Comparing SMRT-Tag and ligation-based libraries, we observed similar recall (0.420 versus 0.527 for SNVs and 0.338 versus 0.408 for indels), precision (0.870 versus 0.898 for SNVs and 0.785 versus 0.797 for indels) and F1 score (0.566 versus 0.664 for SNVs and 0.380 versus 0.539 for indels; Fig. 2c). Performance for SVs was slightly lower (recall 0.129 versus 0.25, precision 0.877 versus 0.879 and F1 score 0.225 versus 0.389; Fig. 2d) probably due to shorter reads affecting the resolution of large indels.

In PacBio SMS, nucleobase modifications are inferred from stereotyped changes in real-time polymerase kinetics during nucleotide addition, offering an opportunity for simultaneous genotyping and epigenotyping^29^. To assess detection of CpG methylation, we predicted the positions of 5-methyl-deoxyctidine (m^5^dC) using PacBio’s primrose software, which assigns methylation probabilities to CpGs via a convolutional neural network that combines kinetic data from multiple CCS passes. We compared primrose methylation calls from SMRT-Tag and ligation-based PacBio SMS against gold standard bisulfite sequencing data^30^. Per-CpG methylation calls were tightly correlated between the SMRT-Tag and bisulfite m^5^dC datasets (Pearson’s *r* = 0.84; Fig. 2e). Framing CpG methylation calling as a classification problem (Fig. 2f), we observed excellent performance measured by the area under the curve (AUC) (Fig. 2g), with the SMRT-Tag and ligation-based datasets demonstrating similar AUC (0.935 versus 0.926, respectively).

Finally, to compare performance at higher depths, we sequenced additional HG002 SMRT-Tag libraries to 11.2× median coverage (34.24 Gb on six Sequel II flow cells). We compared SNV, indel and SV calls from SMRT-Tag and coverage-matched ligation-based libraries against the GIAB HG002 benchmark. We found similar recall (0.970 SMRT-Tag versus 0.970 ligation-based PacBio for SNVs and 0.911 versus 0.907 for indels), precision (0.995 versus 0.995 for SNVs and 0.955 versus 0.949 for indels), F1 score (0.983 versus 0.982 for SNVs and 0.932 versus 0.928 for indels) and AUC (0.969 versus 0.968 for SNVs and 0.902 versus 0.897 for indels; Supplementary Fig. 5a–d). CpG methylation detected using high-coverage SMRT-Tag was on par with short-read bisulfite (Supplementary Fig. 5e) and ligation-based PacBio (Supplementary Fig. 5f) data. SMRT-Tag also resolved variants within segmental duplications, repeats, the major histocompatibility complex locus and other challenging regions (Supplementary Fig. 6a; F1 scores 0.977 SMRT-Tag versus 0.967 ligation-based PacBio for SNVs and 0.912 versus 0.905 for indels across all regions with differences probably due to sequencing chemistry) and at varying levels of coverage (Supplementary Fig. 6b). Taken together, these results demonstrate the strong technical concordance between tagmentation and ligation-based libraries and the sensitive detection of genetic and epigenetic variation by SMRT-Tag.

### Single-fiber chromatin and methylation profiling with SAMOSA-Tag

**Fig. 3:**
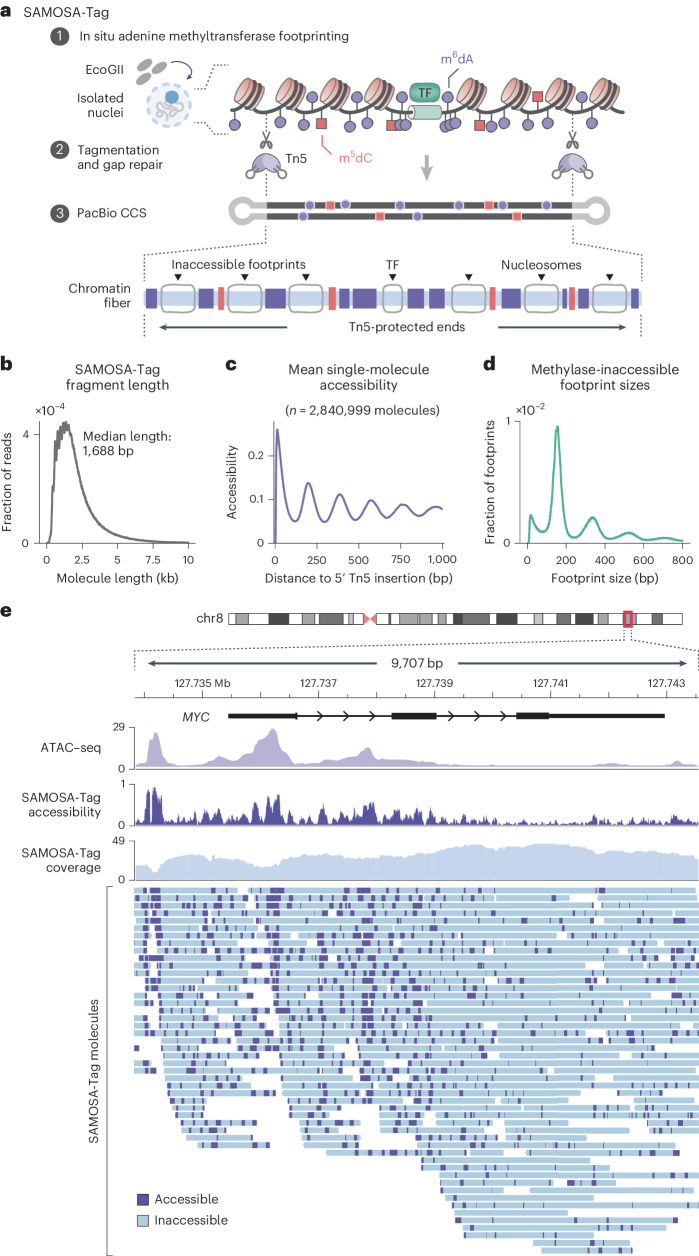
SAMOSA-Tag: single-molecule chromatin profiling via tagmentation of adenine-methylated nuclei. **a**, In SAMOSA-Tag, nuclei were methylated using the non-specific EcoGII m^6^dAase and tagmented in situ with hairpin-loaded transposomes. DNA was purified, gap-repaired and sequenced, resulting in molecules where the ends resulted from Tn5 transposition, the m^6^dA marks represented fiber accessibility and computationally defined unmethylated ‘footprints’ captured protein–DNA interactions. **b**, Length distribution for SAMOSA-Tag molecules from OS152 osteosarcoma cells. **c**,**d**, Average methylation from the first 1-kb of molecules (**c**) and unmethylated footprint size distribution (**d**) for the same data as in **b**. **e**, SAMOSA-Tag fibers at the amplified *MYC* locus. Predicted accessible and inaccessible bases are marked in purple and blue, respectively. Average SAMOSA accessibility is shown in purple; the matched ATAC–seq track is shown in light purple.

Tagmentation is the basis for assay for transposase-accessible chromatin with sequencing (ATAC–seq), a popular method for profiling chromatin accessibility^16^. Reasoning that Tn5 could be used to lower the µg-range input needed for single-molecule chromatin accessibility assays developed by us^4,11^ and others^5,8^, we optimized a tagmentation-assisted SAMOSA (SAMOSA-Tag; Fig. 3a). In SAMOSA-Tag, nuclei are treated in situ with the non-specific EcoGII methyltransferase, which mediates N6-deoxyadenosine methylation (m^6^dA), and tagmented using hairpin-loaded Tn5 under conditions optimal for ATAC–seq^31^. DNA is then purified, gap-repaired and sequenced. As proof of concept, we applied SAMOSA-Tag to 50,000 nuclei from *MYC-*amplified OS152 human osteosarcoma cells^32^ and used a convolutional neural network hidden Markov model^11^ to call inaccessible protein–DNA interaction ‘footprints’ from m^6^dA natively detected by PacBio SMS. In total, we sequenced 3,640,652 molecules (7.79 Gb) across eight replicates. Reflecting the transposition of chromatin in nuclei, SAMOSA-Tag CCS read lengths displayed characteristic oligonucleosomal banding (Fig. 3b). When aligned at the 5′ ends, molecules had periodic accessibility signal, which is consistent with transposition adjacent to nucleosomal barriers (Fig. 3c). Individual single-molecule footprint sizes also corresponded to expected mono-nucleosomes, di-nucleosomes, tri-nucleosomes, etc. (Fig. 3d). Finally, single-fiber accessibility visualized in the genomic context, for example, at the amplified *MYC* locus (Fig. 3e) and at copy number loss and neutral loci (Supplementary Figs. 7 and 8), correlated well with ATAC–seq. Importantly, there was only a mild enrichment of SAMOSA-Tag insertions for transcription start sites (TSS) (Supplementary Fig. 9a). However, insertions tended to occur proximal to predicted CCCTC-binding factor (CTCF) binding sites (Supplementary Fig. 9b), which is consistent with blocked Tn5 transposition by strong barrier elements. This subtle preference was also reflected in the fraction of insertions falling near TSS and CTCF sites (Supplementary Fig. 9c; 1.51-fold and 1.58-fold enrichment above background, respectively) and was consistent with propensities reported for Tn5-based shotgun Illumina libraries^33^. Finally, SAMOSA-Tag generalized well to mouse embryonic stem (ES) cells (Supplementary Fig. 10a–c), recovering characteristic ‘footprints’ around predicted CTCF and REST binding sites, which clustered into distinct accessibility patterns (Supplementary Fig. 10d,e). SAMOSA-Tag can also be performed ex situ wherein DNA is extracted from footprinted nuclei before tagmentation. The barrier effect apparent on aligning 5′-end reads is abrogated in ex situ SAMOSA-Tag (Supplementary Fig. 10b), highlighting the flexibility of the approach for applications requiring more coverage uniformity (Supplementary Note 2).

### SAMOSA-Tag permits integrative single-fiber epigenomics

**Fig. 4:**
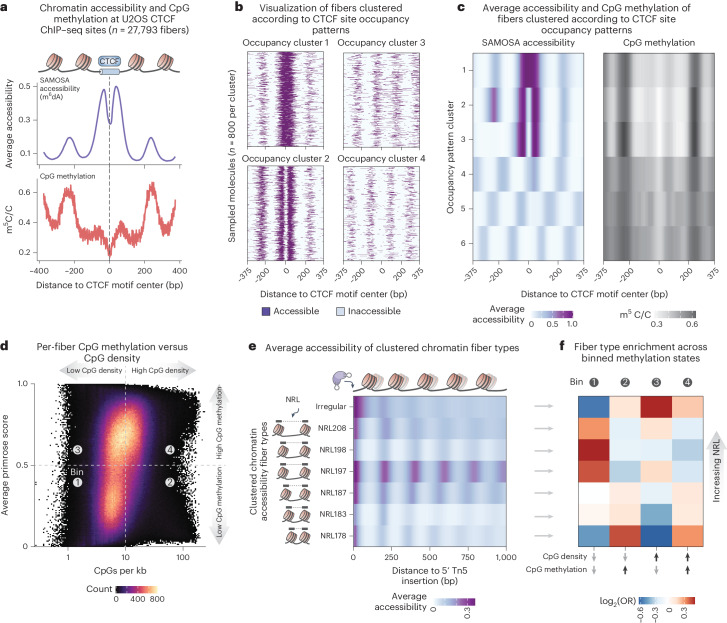
SAMOSA-Tag concurrently profiles protein–DNA interactions and CpG methylation on single chromatin fibers. **a**, Average SAMOSA (m^6^dA) accessibility and CpG methylation on 27,793 footprinted fibers from OS152 human osteosarcoma cells, centered at the binding sites predicted from published U2OS ChIP–seq data^34^. **b**, Visualization of m^6^dA signal for individual, clustered fibers centered at the predicted CTCF motifs, reflecting different CTCF-occupied, accessible and inaccessible states (800 molecules per cluster). **c**, Average accessibility (left) and CpG methylation (right) for each of six clustered accessibility states around CTCF motifs. **a**–**c**, The window size was 750 nt. **d**, Average primrose CpG methylation score for individual fibers as a function of density of CpG dinucleotides per kb. We binned molecules into four classes depending on CpG density and average primrose score. **e**, Average accessibility of seven fiber types determined using Leiden clustering of single-fiber m^6^dA chromatin accessibility autocorrelation. Clusters stratified the entire genome according to NRL (ranging from 178 to 208 nt) or irregularity. **f**, Relative enrichment or depletion of individual fiber types for the same clusters as in **e** in each of four binned states from **d** (one-sided Fisher’s exact test, *P* values ranging from *P* < 2.2 × 10^−308^ to *P* < 2.41 × 10^−5^).

The separability of PacBio polymerase kinetics into m^6^dA and m^5^dC channels affords the opportunity to concurrently ascertain DNA sequence, CpG methylation and single-fiber chromatin accessibility to exogenous adenine methyltransferases in a single assay. We first examined m^6^dA accessibility and CpG methylation at CTCF sites predicted from chromatin immunoprecipitation followed by sequencing (ChIP–seq) in the U2OS osteosarcoma cell line^34^. We recovered hallmarks of CTCF binding, including flanking-positioned nucleosomes, decreased accessibility immediately at the motif (compatible with exclusion of EcoGII by bound CTCF) and depressed CpG methylation within motifs (Fig. 4a). Taking advantage of the single-molecule resolution of SAMOSA-Tag, we deconvolved the differing fiber structures that contribute to the ensemble average chromatin and methylation profiles (Fig. 4a) using Leiden clustering^35^ (see the example of four clusters shown in Fig. 4b; cluster sizes are shown in Supplementary Fig. 11). Analysis of pattern-specific average m^5^dC signal (Fig. 4c) revealed the lowest CpG methylation at CTCF-bound (cluster 1) and unbound and accessible (cluster 2) motif fiber patterns, consistent with previous results^36^. Two additional analyses confirmed minimal confounding of m^5^dC and m^6^dA signals. First, the primrose CpG score distributions of EcoGII untreated negative control and footprinted SAMOSA-Tag libraries were concordant (Supplementary Fig. 12a). Second, the average CpG methylation surrounding predicted CTCF sites on fibers with inaccessible motifs compared with those with footprinted motifs was tightly correlated (Supplementary Fig. 12b).

We previously demonstrated that single-fiber chromatin accessibility data can be used to segment the genome by regularity and average spacing of nucleosomes (nucleosome repeat length (NRL))^4,11^. These studies relied on complementary epigenomic assays to ascertain the distribution of ‘fiber types’ (that is, clusters of molecules with unique regularity or NRL) in euchromatic and heterochromatic domains. We sought to improve on these analyses by directly assessing fiber structure variation with jointly resolved single-molecule CpG content and methylation. To do so, we grouped SAMOSA-Tag molecules into four bins (Fig. 4d) gated on CpG density (>10 CpG dinucleotides per kb) and primrose score (average score greater than 0.5). We then defined fiber types by clustering the m^6^dA accessibility autocorrelation for each molecule 1 kb or longer in length^4,11^. After removing artifactual molecules, we obtained seven distinct clusters (Fig. 4e; cluster sizes are shown in Supplementary Fig. 13) effectively stratifying the OS152 genome according to NRL (clusters NRL178–NRL208) and regularity (irregularity cluster = irregular spacing). Finally, we carried out a series of enrichment tests to assess domain-specific fiber composition across the four CpG content and methylation bins (Fig. 4f; reproducibility shown in Supplementary Fig. 14). We highlight two findings relevant to chromatin regulation: first, putative hypomethylated CpG islands (high CpG content, low CpG methylation) were enriched for fibers that were irregular (odds ratio (OR) for the irregularity cluster = 1.42, *P* < 2.2 × 10^−308^) or have long NRLs (NRL208 OR = 1.09, *P* = 4.43 × 10^−64^; NRL197 OR = 1.11, *P* = 1.49 × 10^−58^); second, probably hypermethylated, CpG-rich repeats (high CpG content, high CpG methylation) were enriched for fibers that were irregular (irregularity cluster OR = 1.14, *P* = 1.33 × 10^−139^) or have short NRLs (NRL172 OR = 1.24; *P* < 2.2 × 10^−308^). These results are consistent with our previous in vivo observations of active promoters and heterochromatin in human cells^4^ and mouse ES cells^11^, pointing to a conserved single-fiber chromatin structure within these domains. Together, these analyses show that SAMOSA-Tag generated multimodal, genome-wide, single-molecule chromatin accessibility data from tens of thousands of cells.

### SAMOSA-Tag of PDXs of patients with prostate cancer

**Fig. 5:**
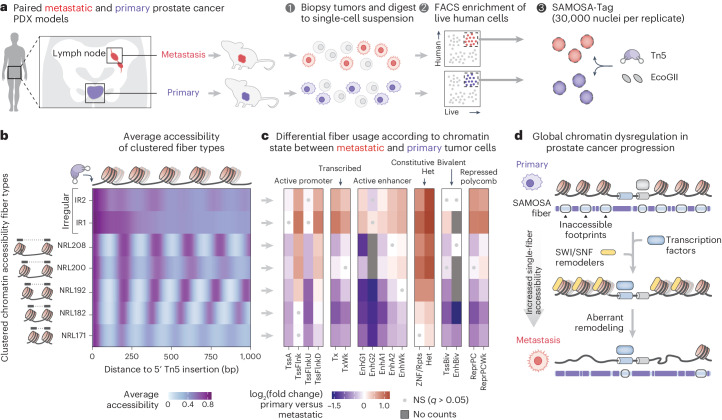
SAMOSA-Tag of PDXs nominates global chromatin dysregulation in prostate cancer metastasis. **a**, Overview of the approach for SAMOSA-Tag of PDXs generated from primary and metastatic castration-resistant prostate tumors sampled from a single patient. Live human cells were enriched from tumors explanted from PDX mice using FACS. Six replicate SAMOSA-Tag reactions were performed using approximately 30,000 nuclei each isolated from primary and metastatic PDXs. **b**, Clustered chromatin fiber types detected in primary and metastatic PDXs falling in one of 17 prostate-specific chromHMM states. Unsupervised Leiden clustering identified seven fiber types—five regular clusters in NRL ranging from 171 to 208 bp and two irregular clusters. **c**, Heatmap of log_2_ fold change in fiber type usage across chromHMM states (∆) in metastatic versus primary PDXs. Effect sizes were the coefficients of case status (primary versus metastatic), which was the predictor variable in a logistic regression model with domain-specific fiber usage as the response variable. Statistically significant differences were identified using a Wald’s test of coefficients; two-tailed *P* values were adjusted for multiple testing using Storey’s *q*^49^, with a significance threshold of *q* ≤ 0.05. Red indicates fiber types enriched in metastasis, while blue indicates fiber types enriched in primary tumors. The gray dots mark non-significant (NS) results. Chromatin state abbreviations: EnhA1 and EnhA2, active enhancers; EnhBiv, bivalent enhancer; EnhG1 and EnhG2, genic enhancers; EnhWk, weak enhancer; ReprPC, repressed polycomb; ReprPCWk, weak repressed polycomb; TssA, active TSS; TssBiv, bivalent/poised TSS; TssFlnk, flanking TSS; TssFlnkD, downstream flanking TSS; TssFlnkU, upstream flanking TSS; Tx, strong transcription; TxWk, weak transcription; ZNF/Rpts, zinc-finger genes and repeats. **d**, Speculative model of changes in single-molecule chromatin accessibility during prostate cancer progression based on PDX SAMOSA-Tag. Highly accessible, irregular chromatin fibers devoid of phased nucleosomes were enriched in metastatic cells, which was suggestive of deranged activity of BAF remodelers, the prime candidates for generating nucleosome-free, irregular, single-molecule accessibility patterns.

One area where SAMOSA-Tag could have immediate utility is in the study of disease models such as cancer PDXs where samples are limited. There are two key challenges with PCR-free PacBio profiling of PDXs propagated in mice: first, after tumor engraftment and growth, cancer cells must be enriched and separated from mouse cells using FACS; second, cells and nuclei from metabolically active or necrotic tumors are often fragile and have damaged native DNA, which impedes sequencing. We thus sought to apply SAMOSA-Tag to generate the first single-fiber chromatin accessibility data from PDX models. We generated PDXs from matched primary and metastatic tumors resected from a patient with castration-resistant prostate cancer^37^, and isolated and footprinted approximately 180,000 nuclei from one mouse each per model (Fig. 5a; the FACS gates are shown in Supplementary Fig. 15). To account for the technical difficulty of working with precious PDX samples while ensuring reproducibility, we opted conservatively to perform six replicate SAMOSA-Tag reactions (approximately 30,000 nuclei per reaction). Primary and metastatic PDX libraries were sequenced to depths of 0.32× (0.95 Gb (22.8%) human alignment) and 0.53× (1.57 Gb (95.9%) human alignment). PDX SAMOSA-Tag had similar technical characteristics to mouse ESCs and the experiments involving OS152 cells (Supplementary Fig. 16). Future optimization of cell enrichment, DNA damage repair and nuclei purification will probably permit higher per-sample coverage using lower input than in the proof of concept presented in this study.

Altered CTCF expression and occupancy have been tied to hyperactive androgen signaling^38^ and prostate cancer progression^39^. To examine single-molecule chromatin accessibility and CTCF binding in primary and metastatic tumor cells (Supplementary Fig. 17a), we clustered PDX SAMOSA-Tag reads aligned to CTCF sites predicted using ENCODE ChIP–seq in LnCaP prostate cancer cells. This revealed multiple clusters (Supplementary Fig. 17b) reflecting varying nucleosome occupancy patterns around the CTCF motif (patterns NO1–NO5), direct CTCF occupancy (pattern A) and ‘hyperaccessible’ fibers devoid of nucleosomes flanking the motif (pattern HA) similar to the OS152 and mouse ESC SAMOSA-Tag (Fig. 4e and Supplementary Fig. 10a). Visualizing differential fiber type usage (Supplementary Fig. 17c) suggested intriguing metastasis-specific shifts in cluster usage, including a decrease in the stereotypic nucleosome phasing at CTCF-bound sites (pattern A) in favor of pattern HA. Analysis of concurrently measured m^5^dC within these clusters suggested subtle preliminary differences in CpG methylation correlated with single-fiber CTCF motif occupancy patterns (Supplementary Fig. 17d).

Finally, we asked whether single-fiber chromatin architecture differed between matched primary and metastatic tumors (Supplementary Fig. 18a). Unsupervised clustering of autocorrelated single-molecule m^6^dA signal from primary and metastatic PDXs yielded six fiber types (Fig. 5b): four regular clusters with NRLs ranging from 171 to 208 bp and two irregular clusters (IR1 and IR2). Using published annotations for healthy human prostate as a reference ^40^, we determined the relative enrichment of fiber types across epigenomic domains (Supplementary Fig. 18b). Applying a logistic regression framework to nominate significant differences in domain-specific fiber usage, we identified several patterns of interest to be followed up in future studies (Fig. 5c). For instance, metastatic tumor cells were significantly enriched for irregular fibers (IR1 and IR2) in heterochromatic domains such as KRAB zinc-finger genes (ZNF/Rpts; IR1 log_2_ fold change (∆) = 0.77, *q* = 7.56 × 10^−7^; IR2 ∆ = 1.03, *q* = 6.15 × 10^−15^) and regions harboring marks of constitutive heterochromatin (Het) IR1 ∆ = 1.22, *q* = 1.45 × 10^−177^; IR2 ∆ = 1.25; *q* = 4.46 × 10^−125^). In contrast, distal enhancers were significantly depleted for fibers with specific NRLs (for example, active enhancer 1 (EnhA1); NRL182 ∆ = −1.11, *q* = 1.07 × 10^−71^). These data hint at the involvement of ATP-dependent chromatin remodelers, such as the Brahma-associated factor (BAF) complex in metastasis-associated nucleosome eviction and chromatin disorganization (Fig. 5d). While BAF has already been implicated as a driver of prostate cancer progression^41^, mechanistic studies are needed to evaluate the proposed model. Taken together, these data demonstrate the potential of SAMOSA-Tag to yield biological insights in challenging disease models.

## Discussion

We optimized direct Tn5 transposition of hairpin adapters as a general strategy for preparing amplification-free, multiplexable PacBio libraries from limiting amounts of input DNA. We applied this principle to develop two methods that take advantage of the simultaneous readout of modified and unmodified bases using SMS and highlight the broad potential of Tn5-based PacBio library generation. First, tagmentation coupled with PacBio HiFi sequencing (SMRT-Tag) allowed detection of genetic variation and CpG methylation from as little as 40 ng gDNA (approximately 7,000 human cells) with accuracy comparable to conventional whole-genome and bisulfite sequencing. Second, tagmentation of 30,000–50,000 nuclei after adenine methyltransferase chromatin footprinting (SAMOSA-Tag) concurrently resolved single-fiber DNA sequence, CpG methylation and chromatin accessibility in one assay. Using SAMOSA-Tag libraries multiplexed to maximize sequencing yield, we resolved CTCF binding, nucleosome architecture and CpG methylation in osteosarcoma cells. We also carried out single-molecule epigenome analyses in a preclinical disease model, uncovering global chromatin dysregulation associated with metastatic progression in technically challenging prostate cancer PDX cells.

We anticipate that tagmentation-based protocols will address several obstacles to single-molecule genomics. Simplification of library preparation by combining DNA fragmentation and adapter ligation steps and the high efficiency of Tn5 transposition permitted 90–99% input reduction for SMRT-Tag and SAMOSA-Tag, placing sequencing at the lower limit of the PacBio platform within reach (Supplementary Notes 1 and 2). The ability to profile unamplified DNA has implications for basic and translational analyses of rare cell populations that integrate the breadth of nucleotide, structural and epigenomic variation captured natively by SMS without chemical conversion. Importantly, in situ tagmentation also obviates the need for DNA purification, raising the exciting prospect of multimodal genomics with both single-cell and single-molecule resolution. We envision that developments such as droplet-based or combinatorial barcoding-based cellular indexing^21,23,42^ will extend massively parallel PCR-free, single-molecule assays to individual cells for applications ranging from strand-specific somatic variant detection^43^ to haplotype-resolved de novo assembly, and cell type classification.

As with any technical advance, while SMRT-Tag and SAMOSA-Tag illustrate the power of Tn5 transposition for PacBio SMS, they have several limitations and areas for improvement. Because these methods do not rely on PCR, libraries may need to be multiplexed to maximize OPLC and reduce per-base cost. Still, we showed that flow cells can be efficiently loaded with as little as 40 ng starting input mass. The length of molecules is lower than the 15–20-kb capability of PacBio SMS and is primarily controlled by transposome concentration and optional bead-based size selection; the limited input amount precludes gel-based-size fractionation. Furthermore, the inverse proportionality between length and molarity for a given input mass implies that more starting material or pooling at higher plexity are needed to yield deep coverage (Supplementary Note 2). This is salient for comprehensively surveying variants and particularly for SV discovery because breakpoint-spanning molecules are less abundant in SMRT-Tag than ligation-based libraries. Limited coverage may also impede resolution of epigenomes in tissues and other heterogeneous samples. Although we have partially addressed this by demonstrating the tunability of tagmentation, adapting engineered^25^ and bead-linked^44^ transposases may offer finer control of molecule length in the future. SAMOSA-Tag is also limited by the minimum number of nuclei that can be processed. In this study, we generated high-quality data from replicates of 30,000–50,000 nuclei. Optimizations including mild fixation, miniaturized methylation reactions or immobilization of nuclei on beads^45^ could further relax this constraint.

More generally, SMRT-Tag and SAMOSA-Tag add to a growing series of innovations centered around third-generation sequencing, including Cas9-targeted sequence capture^46^, combinatorial indexing-based plasmid reconstruction^47^ and concatenation-based isoform-resolved transcriptomics^48^. The widespread adoption of short-read genomics in basic and clinical applications, and the transition from bulk to single-cell assays was catalyzed by approaches that simplified library preparation and reduced input requirement. Direct transposition offers similar promise for rapidly maturing third-generation sequencing technologies in enabling scalable, sensitive and high-fidelity telomere–telomere genomics and epigenomics.

## Methods

### Human patient and animal studies

De-identified primary tumor and metastatic lymph node tissue used to generate the PDX models was donated by a patient who provided written informed consent under protocol no. 90911 ‘Use of marker in cytometric analysis in prostate cancer to predict biological potential’ (University of California, San Francisco (UCSF) institutional review board 11-05226). NOD *scid* gamma (NSG) mice (UCSF Breeding Core) were maintained under pathogen-free conditions. This study was performed with assistance from the UCSF Laboratory Animal Resource Center under a protocol approved by the UCSF Institutional Animal Care and Use Committee (no. AN195508).

### Cell lines

OS152 cells were regularly tested for authenticity and *Mycoplasma* via CellCheck 9 Plus (IDEXX BioAnalytics) and were cultured in DMEM (catalog no. 10-013-CV, Corning) with 10% Bovine Growth Serum (catalog no. SH30541.03, HyClone) and 1% 100× penicillin-streptomycin-glutamine (catalog no. 30-009-CI, Corning). Embryonic day 14 (E14) mouse ES cells were a gift from E. Nora (UCSF) and were routinely tested for *Mycoplasma* via PCR (NEBNext Ultra II Q5 Master Mix, catalog no. M0544S, New England Biolabs). Feeder-free cultures were passaged at least twice before use and maintained on 0.2% gelatin and in KnockOut DMEM (catalog no. 10829018, Thermo Fisher Scientific) with 10% FCS (catalog no. BW-067C18, Phoenix Scientific), 1% GlutaMAX (catalog no. 35050061, Thermo Fisher Scientific), 1% MEM Non-Essential Amino Acids Solution (catalog no. 1114050, Thermo Fisher Scientific), 0.128 mM 2-mercaptoethanol (catalog no. 1610710XTU, Bio-Rad Laboratories) and purified 1× leukemia inhibitory factor (gifted by B. Panning, UCSF).

### Assembly of hairpin adapter-loaded Tn5 transposomes

#### Annealing adapters

Uniquely barcoded (Hamming distance ≥4), HPLC-purified hairpin oligonucleotides (Supplementary Table 5) were purchased from Integrated DNA Technologies and normalized to 100 µM in nuclease-free water. Adapters were diluted to 20 µM in annealing buffer (10 mM Tris-HCl, pH 7.5, and 100 mM NaCl), annealed (95 °C for 5 min, 25 °C for 30 min, held at 4 °C) and stored at −20 °C.

#### Loading Tn5 with SMRT-Tag adapters

Triple-mutant Tn5^R27S,E54K,L372P^ enzyme (Tn5) was purified by the QB3 MacroLab (University of California, Berkeley). Aliquots of Tn5 (3.9 mg ml^−1^) in storage buffer (50 mM Tris-HCl, pH 7.5, 800 mM NaCl, 0.2 mM EDTA, 2 mM dithiothreitol, 10% glycerol) and frozen at −20 °C were thawed at 4 °C, diluted in dilution buffer (50 mM Tris-HCl, pH 7.5, 200 mM NaCl, 0.1 mM EDTA, 2 mM dithiothreitol, 50% glycerol) to approximately 1 mg ml^−^^1^ Tn5 (18.9-µM monomer) by mixing at 4 °C for 3.5 h until homogenized. Hairpin adapters were loaded by mixing 1.02× volumes of 1 mg ml^−1^ Tn5 with 1× volume of 20 µM annealed adapters, followed by incubation at 23 °C with agitation at 350 r.p.m. for 55 min. Loaded Tn5 stock (9.4-µM monomer) was stored at −20 °C for up to 6 months in glycerol to a final concentration of 50%. Then, 1–2 µl of loaded Tn5 diluted in NativePAGE Loading Buffer was run on a NativePAGE 4–16% Bis-Tris gel (catalog nos. BN2003 and BN1002, Thermo Fisher Scientific) at 150 V for 1 h at 4 °C followed by 180 V for 15 min. Gels were stained with SYBR Gold (catalog no. S11494, Thermo Fisher Scientific) in Tris base, acetic acid and EDTA buffer and then SimplyBlue (catalog no. LC6060, Thermo Fisher Scientific) for 1 h and imaged on an Odyssey XF Imaging System (software v.1.1.0.61, LI-COR).

#### Assessing the tunability of fragment lengths

Serial dilutions of transposome stock were incubated with 160 ng human gDNA (Promega Corporation) while varying buffers, temperatures and incubation times. Analytical electrophoresis was performed on a 0.4–0.6% Tris base, acetic acid and EDTA agarose gel run at 60–80 °C for 2–3 h. Gels were stained with SYBR Gold and imaged on an Odyssey XF Imaging System.

### SMRT-Tag of gDNA

#### SMRT-Tag library preparation

HG002, HG003 and HG004 gDNAs (Coriell Institute for Medical Research) normalized to 40–160 ng in 9 µl Tagmentation Mix (10 mM [tris(hydroxymethyl)methylamino]propanesulfonic acid-NaOH, pH 8.5, 5 mM MgCl_2_, 10% dimethylformamide) were tagmented with 1 µl of barcoded Tn5 at varying concentrations (Supplementary Table 3) at 55 °C for 30 min. Reactions were terminated by adding 0.2% SDS (final concentration 0.04%) before incubation at 25 °C for 5 min, 2× SPRI cleanup and elution in 12 µl elution buffer (EB) (10 mM Tris-HCl, pH 8.5). DNA was gap-repaired at 37 °C for 1 h in repair mix (2 U Phusion-HF, 80 U Taq DNA Ligase with 1× Taq DNA ligase buffer and 0.8 mM deoxynucleotide triphosphate; catalog nos. M0530S, M0208S and N0447S, New England Biolabs), purified with 2× SPRI beads, eluted in 12 µl EB and digested at 37 °C for 1 h in ExoDigest Mix (100 U exonuclease III per 160 ng DNA, 1× NEBuffer 2, catalog nos. M0206S and B7002S, New England Biolabs). Libraries were eluted in 12 µl EB after 2× SPRI cleanup.

#### Titration of transposome and input amounts at varying temperatures

To characterize the tunability of tagmentation, reactions were carried out as above using hairpin-loaded Tn5 stock (9.4-μM monomer) diluted in nuclease-free water to 0.05-pmol, 0.50-pmol and 5-pmol monomers with 40, 200 and 1,000 ng HG003 gDNA and incubated at 37 °C or 55 °C for 30 min.

#### Assaying barcode hopping via pooled gap repair

Libraries were prepared as above using barcoded transposomes, but were pooled after tagmentation into a single repair reaction before exonuclease digestion.

#### Optional size selection of libraries

Size selection was performed to enrich for molecules larger than 5 kb (high-molecular weight molecules) by binding libraries to 3.1× volumes of 35% (v/v) AMPure PB beads (catalog no. 100-265-900, PacBio) diluted in EB at 25 °C for 15 min. Beads were washed twice with 80% ethanol for 1 min and DNA was eluted in 15 µl EB. For some libraries, 0.25× AMPure PB cleanup of the supernatant was used to recover low-molecular weight (<5-kb) DNA, which was eluted in 15 µl EB.

### SAMOSA-Tag of cell lines

#### Nuclei isolation

A total of 1–2 × 10^6^ OS152 or mouse E14 ES cells were collected by centrifugation (300*g*, 4 °C, 10 min), washed in cold PBS and resuspended using a wide-bore micropipette tip in 1 ml cold nuclei lysis buffer (20 mM HEPES, 10 mM KCl, 1 mM MgCl_2_, 0.1% Triton X-100, 20% glycerol, 1× protease inhibitor (catalog no. 04693132001, Roche)). Cells were lysed on ice for 5 min. Nuclei were pelleted (600*g*, 4 °C, 10 min), washed with Buffer M (15 mM Tris-HCl, pH 8.0, 15 mM NaCl, 60 mM KCl, 0.5 mM spermidine) and counted on a Countess 3 instrument (Thermo Fisher Scientific).

#### SAMOSA footprinting

Permeabilized nuclei were pelleted (600*g*, 4 °C, 10 min) and resuspended in 400 µl Buffer M with 1 mM *S*-adenosyl methionine (catalog no. B9003S, New England Biolabs); 200 µl was reserved as an unmethylated control. Nuclei were treated with 250 U EcoGII (25,000 U ml^−1^; New England Biolabs) for 30 min at 37 °C with agitation at 300 r.p.m. every 2 min. *S*-adenosyl methionine was replenished to 1.16 mM after 15 min in the methylation and control reactions. Nuclei were pelleted (600*g*, 10 min, 25 °C) and resuspended in 250 µl Omni-ATAC Buffer (10 mM Tris-HCl, pH 7.5, 5 mM MgCl_2_, 0.33× PBS, 10% dimethylformamide, 0.01% digitonin, 0.1% Tween 20). Nuclei were filtered through a Flowmi 40-µm strainer (catalog no. BAH136800040, Sigma-Aldrich) and counted and visualized on a Countess 3 to verify dissociation of aggregates.

#### In situ SAMOSA-Tag

Methylated and unmethylated samples were split into 10,000–50,000 nuclei aliquots and, based on the desired library size and cell type, 9.4–18.8 pmol barcoded transposome was added. Samples were brought up to 50 µl with Omni-ATAC Buffer before tagmentation at 55 °C for 45–60 min. Tagmentation was terminated by treating nuclei with 10 µg RNase A (catalog no. EN0531, Thermo Fisher Scientific) at 37 °C for 15 min with 300 r.p.m. shaking, mixing with 50 µg proteinase K (catalog no. AM2546, Thermo Fisher Scientific), 2.5 µl 10% SDS and 2.5 µl of 0.5 M EDTA, and incubation at 60 °C with 1,000 r.p.m. shaking for 1–2 h. Tagmented DNA was bound to 2× SPRI beads at 23 °C for 30 min with mixing at 350 r.p.m. every 3 min. Beads were collected on a magnet and washed twice in 80% ethanol for 1 min before elution in 20 µl EB at 37 °C for 15 min with mixing at 350 r.p.m. every 3 min. An additional 0.6× SPRI cleanup was used to enrich for fragments larger than 500 bp. DNA was stored at 4 °C overnight, or up to 2 weeks at −20 °C. Libraries were prepared by incubating DNA in Repair Mix at 37 °C for 1 h, followed by 2× SPRI cleanup, treatment with ExoDigest Mix at 37 °C for 1 h, 2× SPRI cleanup and elution in 12 µl EB. For OS152 and mouse E14 ES SAMOSA-Tag cells, eight methylated and unmethylated control replicates were tagmented with barcoded Tn5.

#### Ex situ SAMOSA-Tag

Permeabilized mouse E14 ES cell nuclei were footprinted as above. After EcoGII methylation, nuclei were digested with 10 µg RNase A at 37 °C for 15 min, mixed with 2.65 µl 10% SDS, approximately 50 µg proteinase K and incubated at 65 °C for 3 h. DNA was extracted in 1× volume of phenol:chloroform:isoamyl alcohol (25:24:1, v/v). Samples were centrifuged (16,000*g*, 2 min, 25 °C) and the aqueous phase was mixed with a 0.1× volume of 3 M NaOAc, 1 µl GlycoBlue Coprecipitant (catalog no. AM9515, Thermo Fisher Scientific) and 3× volumes of cold 100% ethanol. DNA precipitated overnight at −80°C was then pelleted (16,000*g*, 30 min, 4 °C), washed with 500 µl 70% ethanol, air-dried and resuspended in 40 µl EB. Then, 100 ng SAMOSA DNA was tagmented with a 0.046-pmol Tn5 monomer with library preparation as described for SMRT-Tag.

### SAMOSA-Tag of PDXs

#### Generation of prostate cancer PDXs

PDXs were generated^37^ using 3–5-mm primary tumor and synchronous lymph node metastasis fragments isolated from a 71-year-old male who presented with high-risk prostate cancer (pretreatment prostate-specific antigen = 19.1 ng ml^−1^, Gleason 4+5, T3aN1M0) with 6–9-mm bilateral external pelvic lymph node metastases on prostate-specific membrane antigen positron emission tomography scan. To minimize cell death and preserve microenvironment integrity, tumor fragments were taken immediately after prostatic devascularization during robotic prostatectomy and pelvic lymph node dissection, placed in 10 ml of Roswell Park Memorial Institute 1640 and implanted subcutaneously into the flanks of 6–8-week-old male NSG mice to establish the PDX lines. PDXs were cryopreserved after three passages in mice. To ensure that PDXs faithfully recapitulated the original tumor and heterogeneity of prostate cancer, sections were subjected to histopathological comparison after each passage and growth patterns were examined. Passage 10 PDXs were used for SAMOSA-Tag.

#### PDX processing and SAMOSA-Tag

Surgically explanted tumors from PDX mice were immediately placed into sterile Roswell Park Memorial Institute 1640 on ice and minced with a scalpel. PDXs were dissociated into single cells by digestion with 10 µg DNase I and 65 mg Collagenase Type 3 (DNase I, RNase & Protease Free, catalog no. LS004206, Worthington Biochemical) and 10 mg Liberase TL (catalog no. 05401020001, Sigma-Aldrich) at 37 °C for 1 h with agitation at 750 r.p.m. in Digestion Buffer: 5 ml DMEM, 5 ml Ham’s F-12, 100 µl 100× penicillin-streptomycin and 40 µl 0.25 mg ml^−1^ Amphotericin B (catalog nos. 11965092, 11765054 and 15290018, respectively, Thermo Fisher Scientific). Cells were centrifuged (800*g*, 5 min, 4 °C), resuspended in 1 ml cold PBS, strained through a Falcon 70-µm strainer (catalog no. 352350, Corning) with a wide-bore micropipette tip, washed twice in 1 ml cold PBS and resuspended in 1 ml Cell Staining Buffer (catalog no. 420201, BioLegend) to approximately 8–12.5 × 10^6^ cells per ml. Cells were blocked with 20 µl Human TruStain FcX Receptor Blocking Solution (catalog no. 422301, BioLegend) for 10 min at 4 °C, stained with PE anti-mouse H-2 antibody (1 µg antibody per 8–12.5 × 10^6^ cells, catalog no. 125505, BioLegend) for 25 min at 4 °C in the dark, washed twice in Cell Staining Buffer and pelleted at 350*g* and 4 °C. Cells were then stained with 1 µl SYTOX Red Dead Cell Stain (catalog no. S34859, Thermo Fisher Scientific) for 15 min at 4 °C. Mouse and dead human cells were depleted using a FACSAria II (FACSDiva software v.9.0.1, BD Biosciences) at the UCSF Center for Advanced Technology. Live human cells were selected as PE^−^ and APC^−^ (SYTOX Red) from singlets gated on forward scatter and collected into a conical tube containing 1 ml PBS. The yield per PDX was 1.20–1.75 × 10^6^ cells. In situ SAMOSA-Tag was performed using sorted cells with spin speed reduced to 400*g*. Limited unmethylated control replicates for primary (*n* = 2) and metastatic (*n* = 1) PDXs were performed due to sample losses.

### Ligation-based library preparation

#### Preparation of low-input libraries

Replicate libraries were prepared from 40 ng sheared HG002 gDNA using the SMRTbell Express Template Prep Kit 2.0 (TPK2.0, catalog no. 101-696-100, PacBio) protocol, which involves overhang removal, DNA damage repair, end repair, A-tailing, adapter ligation, exonuclease digestion and 1× AMPure PB cleanup. Insufficient mass was obtained for sequencing.

#### Preparation of high-input libraries

Phenol:chloroform:isoamyl alcohol-extracted mouse E14 ES cell gDNA was fragmented to 6–8 kb using a g-TUBE (catalog no. 520079, Covaris) with an Eppendorf 5424 rotor spun at 7,000 r.p.m. for six passes. A TPK2.0 library was prepared from 2.5 µg sheared DNA and loaded at 44.6 pM to confirm sequencing of ligation-based libraries at low OPLC.

### DNA quality control and PacBio sequencing

To assess repair efficiency, 1 µl of library before and after exonuclease digestion was quantified using a Qubit 1× High Sensitivity DNA Assay (catalog no. Q33230, Thermo Fisher Scientific). To validate library concentration and size, 1 µl of library was analyzed using Qubit 1× High Sensitivity DNA and Agilent 2100 Bioanalyzer High Sensitivity DNA assays. Sequencing was performed using 8 M SMRTcells (catalog no. 101-389-001, PacBio) on a PacBio Sequel II running SMRTlink v.11.0.0.146107; movies were collected for 30 h with 2-h pre-extension and 4-h immobilization times. Both 2.1 and 2.2 polymerases were used for SMRT-Tag and OS152, and mouse E14 ES cell SAMOSA-Tag, depending on library size (for example, low-molecular weight and high-molecular weight SMRT-Tag libraries were sequenced with 2.1 and 2.2 polymerases, respectively; Supplementary Note 2). PDX SAMOSA-Tag libraries were multiplexed and sequenced using 2.1 polymerase.

### Data analyses

#### Reaction efficiency

Stepwise tagmentation, gap repair and exonuclease efficiencies were defined as the mass ratio of output to input DNA for a given step. The term ‘repair efficiency’ refers to exonuclease cleanup efficiency, as a proxy for effectiveness of gap repair and conversion of DNA into sequenceable molecules. Overall efficiency was estimated as the mass ratio of final library to input DNA, or as the product of stepwise efficiencies.

#### Data preprocessing

CCS/HiFi reads were generated from subreads using ccs v.6.4.0 (PacBio) with the flag --hifi-kinetics. Lima v.2.6.0 (PacBio) with FLAG --ccs was used for demultiplexing and pbmerge v.1.0.0 (PacBio) was used to combine data from libraries sequenced on multiple flow cells. Reads were aligned using pbmm2 v.1.9.0 (PacBio) to the following references: hs37d5 GRCh37 for SMRT-Tag variant analyses; hg38 for OS152 SAMOSA-Tag and all other SMRT-Tag analyses; GRCm38 for mouse E14 ES SAMOSA-Tag cells; and a joint hg38/GRCm39 reference for PDX SAMOSA-Tag with reads uniquely aligned to hg38 retained for subsequent analyses. Read quality was ascertained from ccs and the empiric quality score (Q-score) was calculated as −log_10_(1 − (*n*_matches_ */* (*n*_matches_ *+* *n*_mismatches_ *+* *n*_del_ *+* *n*_ins_))) or the maximal theoretical quality score if the read contained no variation.

#### Analysis of SMRT-Tag demultiplexing

Given the low coverage, SNVs were called naively using SAMtools mpileup (v.1.15.1) in GIAB benchmark intervals supported by two or more reads. For each individual, SNV calls were intersected with private SNVs in regions labeled ‘not difficult’ in the GIAB v.3.0 stratification^25^ (Supplementary Methods).

#### HG002 variant calling and benchmarking

HG002 SMRT-Tag and GIAB PacBio data were subsampled to threefold, fivefold, tenfold and 15-fold depths (Supplementary Methods). SNVs and indels called using DeepVariant (v.1.4.0) were compared against the GIAB/NIST v.4.2.1 benchmarks^27^ using hap.py (v.0.3.12) and GIAB GRCh37 stratifications (v.3.0). SVs called using pbsv v.2.8.0 (PacBio) were compared against NIST Tier 1 HG002 SV calls (v.0.6) using Truvari^50^ (v.3.3.0).

#### Predicting CpG methylation in PacBio reads

PacBio primrose v.1.3.0 (now Jasmine) was used to predict CpG methylation. Methylation probabilities encoded in the BAM tags ML and MM were parsed to continuous values for single-molecule methylation prediction. Per-CpG methylation was estimated using the tools available at github.com/PacificBiosciences/pb-CpG-tools.

#### SAMOSA footprinting

A series of neural networks trained on per-read polymerization kinetics measured during PacBio sequencing of methylated and unmethylated controls were used to predict strand-specific m^6^dA methylation probabilities on SAMOSA-Tag CCS reads^4,11^. Probabilities were binarized into accessibility calls using a two-state hidden Markov model and encoded in the MM and ML BAM tags^4,11^.

#### Comparing ATAC–seq and SAMOSA-Tag

SAMOSA accessibility and the normalized ATAC–seq signal were aggregated at OS152 ATAC–seq peaks. Pearson’s *r* was used to correlate log-transformed values.

#### U2OS and LNCaP CTCF ChIP–seq processing

CTCF binding sites were determined^4^ from published ChIP–seq peaks from U2OS^34^ and LNCaP metastatic prostate adenocarcinoma^51^ cells lifted over from hg19 to hg38.

#### Insertion preference analyses at TSS and CTCF sites

SAMOSA-Tag read ends were tabulated in 5-kb windows around GENCODE v.28 (hg38) or M25 (GRCm38) TSS or ChIP–seq-backed CTCF motifs. Meta-plots were smoothed with a 100-nt running mean. FRITSS and FRICBS were calculated as the fraction of ends falling within the 5-kb window.

#### CTCF CpG and accessibility analyses

The single-fiber m^6^dA accessibility signal around predicted CTCF sites was subjected to Leiden clustering^35^. Clusters comprising less than 10% of data were removed. Unmethylated SAMOSA-Tag fibers were also removed (*n* = 3,627 or 11.5% of all CTCF-motif-containing fibers in OS152; and *n* = 245 or 1.5% in PDX).

#### Classifying fibers according to CpG content and CpG methylation

Fibers were binned according to CpG content (CpGs per kb ≤10 (low) versus >10 (high)) and methylation (average primrose score ≤0.5 (low) versus >0.5 (high)) to define four classes: high CpG content and high methylation; low CpG content and low methylation; high CpG content and low methylation; and low CpG content and high methylation.

#### Fiber type clustering and enrichment

Unsupervised Leiden clustering^35^ of single-molecule accessibility autocorrelation^4,11^ was used to identify fiber types. Clusters comprising less than 10% of all fibers were filtered out; unmethylated or lowly methylated molecules were also removed in the OS152 SAMOSA-Tag analyses (*n* = 317,768 or 12.5% of fibers). For fiber type A stratified according to feature B, a contingency table of fiber counts in four groups (A ∩ B, A ∩ B, A ‘ ∩ B and A ‘ ∩ B ‘ where the complement of set A is denoted by A') was used as input for a one-sided Fisher’s exact test. *P* values were corrected for multiple testing using Storey’s *q*^49^.

#### Differential fiber use

chromHMM domains in normal prostate^40^ were lifted over from hg19 to hg38. Fiber type-stratified coverage of prostate-specific epigenomic domains was tabulated by aggregating the counts of fiber type A mapping to domain B (A ∩ B) versus the other domains (A ∩ B’) per replicate. Counts were normalized across replicates using a median-of-medians approach to account for depth and used as weights for a logistic regression model with domain and fiber status, and case status (primary versus metastasis), as the response and predictor variables, respectively. The glm function (R v.4.2.1) was used to fit the model; the null model was fitted with coefficients set to zero. The case status coefficient was taken to estimate the log_2_ fold change (∆) in metastatic versus primary PDX. This was repeated for all observed combinations of the seven fiber types and 17 domains. A Wald’s test was used to evaluate the maximum likelihood-fitted coefficients; two-sided *P* values were adjusted for multiple testing using Storey’s *q*^49^, with a significance threshold of *q* ≤ 0.05.

#### Visualization

Plots were created using R (v.4.2.1) and ggplot2 (ggplot2.tidyverse.org/). The SAMOSA-Tag data were visualized using a modified version of IGV v.2.17.3 (github.com/RamaniLab/SMRT-Tag/tree/main/igv-vis). The FACS plots were created in FlowJo v.10.8.2 (BD Biosciences).

### Reporting summary

Further information on research design is available in the Nature Portfolio Reporting Summary linked to this article.

## Online content

Any methods, additional references, Nature Portfolio reporting summaries, source data, extended data, supplementary information, acknowledgements, peer review information; details of author contributions and competing interests; and statements of data and code availability are available at 10.1038/s41588-024-01748-0.

### Supplementary information

Supplementary Information Supplementary Methods, Notes 1 and 2, References and Figs. 1–19.

Reporting Summary

Supplementary Table 1 Supplementary Table 1 Repair conditions tested for optimization of gap repair

Supplementary Table 2 Supplementary Table 2 Efficiencies for selected gap repair reactions

Supplementary Table 3 Supplementary Table 3 Summary and sequencing characteristics of the SMRT-Tag, SAMOSA-Tag and TPK2.0 libraries generated in this study

Supplementary Table 4 Supplementary Table 4 Overview of libraries and pooling strategy for the analyses reported in the main and supplementary figures

Supplementary Table 5 Supplementary Table 5 Barcode sequences

### Source data

Source Data Fig. 1 Unprocessed analytical agarose gel for Fig. 1b.

## Data Availability

The SMRT-Tag data are deposited in the NCBI Sequence Read Archive (SRA) under accession no. PRJNA863422. The OS152 and mouse ES cell SAMOSA-Tag data, including the subreads and kinetic parameters, are deposited in the Gene Expression Omnibus (GEO) under accession no. GSE225314. The PDX SAMOSA-Tag data are available via the controlled access database of Genotypes and Phenotypes repository (study accession no. phs003511.v1). The following reference genome assemblies or annotations were used in this study: hs37d5 GRCh37; hg38 GRCm38; a concatenated hg38/GRCm39 reference; GENCODE v.28 and M25; and UCSC hg19 tandem repeats. The NIST/GIAB GRCh37 genome stratifications (v.3.0), small variant benchmarks for HG002, HG003 and HG004 (v.4.2.1), and Tier 1 SV calls for HG002 (v.0.6) were obtained from the NCBI (Supplementary Methods). The following additional publicly accessible datasets were used: GIAB-generated PacBio Sequel II HiFi reads from HG002 (SRA accession no. SRX5527202); CpG methylation calls from bisulfite sequencing of HG002 (ONT Benchmark Datasets; Supplementary Methods); CTCF binding sites determined by ChIP–seq in U2OS (GEO accession no. GSE87831); LNCaP (ENCODE accession no. ENCFF275GDH) cells; and chromHMM annotations for normal prostate (National Genomics Data Center accession no. OMIX237-64-02). Source data are provided with this paper.

## Code availability

The source code used to perform the analyses and step-by-step SMRT-Tag and SAMOSA-Tag protocols is publicly accessible via GitHub at github.com/RamaniLab/SMRT-Tag and *Zenodo* (10.5281/zenodo.10933181)^52^.
