# Supplementary material for: Direct transposition of native DNA for sensitive multimodal single-molecule sequencing
Source: Nat Genet. 2024 May 9;56(6):1300–9. doi: 10.1038/s41588-024-01748-0 (PMC11176058; doi:10.1038/s41588-024-01748-0)
Supplement: Supplementary file 1 — Supplementary Methods, Notes 1 and 2, References and Figs. 1–19. [file 41588_2024_1748_MOESM1_ESM.pdf]

nature genetics

Technical Report

https://doi.org/10.1038/s41588-024-01748-0

# Direct transposition of native DNA for sensitive multimodal single-molecule sequencing

In the format provided by the authors and unedited

## SUPPLEMENTARY METHODS

### SNV-based analysis of SMRT-Tag demultiplexing

The hs37d5 GRCh37 reference genome, GIAB v4.2.1 benchmark VCF and BED files for HG002, HG003, and HG004, and GIAB v3.0 GRCh37 genome stratifications[1] were accessed via the following links:

```
ftp://ftp-trace.ncbi.nlm.nih.gov/giab/ftp/release/references/GRCh37/hs37d5.fa.gz
ftp://ftp-
trace.ncbi.nlm.nih.gov/giab/ftp/release/AshkenazimTrio/HG002_NA24385_son/NISTv4.2.1/GRCh37/
ftp://ftp-
trace.ncbi.nlm.nih.gov/giab/ftp/release/AshkenazimTrio/HG003_NA24149_father/NISTv4.2.1/GRCh37/
ftp://ftp-
trace.ncbi.nlm.nih.gov/giab/ftp/release/AshkenazimTrio/HG004_NA24143_mother/NISTv4.2.1/GRCh37/
ftp://ftp-trace.ncbi.nlm.nih.gov/giab/ftp/release/genome-stratifications/v3.0/v3.0-
stratifications-GRCh37.tar.gz
```

Private SNVs for each individual were obtained using *bcftools* (v1.15.1) and regions for variant calling/evaluation comprising the union of the benchmark BED files were generated using *bedtools* (v2.30.0):

```
bcftools isec --threads 4 -n~100 -w 1 -c some \
    -Oz -o unique.HG002_GRCh37_1_22_v4.2.1_benchmark.vcf.gz \
    HG002_GRCh37_1_22_v4.2.1_benchmark.vcf.gz \
    HG003_GRCh37_1_22_v4.2.1_benchmark.vcf.gz \
    HG004_GRCh37_1_22_v4.2.1_benchmark.vcf.gz

bcftools isec --threads 4 -n~010 -w 2 -c some \
    -Oz -o unique.HG003_GRCh37_1_22_v4.2.1_benchmark.vcf.gz \
    HG002_GRCh37_1_22_v4.2.1_benchmark.vcf.gz \
    HG003_GRCh37_1_22_v4.2.1_benchmark.vcf.gz \
    HG004_GRCh37_1_22_v4.2.1_benchmark.vcf.gz

bcftools isec --threads 4 -n~001 -w 3 -c some \
    -Oz -o unique.HG004_GRCh37_1_22_v4.2.1_benchmark.vcf.gz \
    HG002_GRCh37_1_22_v4.2.1_benchmark.vcf.gz \
    HG003_GRCh37_1_22_v4.2.1_benchmark.vcf.gz \
    HG004_GRCh37_1_22_v4.2.1_benchmark.vcf.gz

cat HG002_GRCh37_1_22_v4.2.1_benchmark_noinconsistent.bed \
   HG003_GRCh37_1_22_v4.2.1_benchmark_noinconsistent.bed \
   HG004_GRCh37_1_22_v4.2.1_benchmark_noinconsistent.bed | \
sort -k1,1 -k2,2n -k3,3n | \
bedtools merge | bgzip | > HG002-4.calling_regions.bed.gz
```

Demultiplexed HG002, HG003, and HG004 SMRT-Tag reads were aligned to hs37d5 using the *minimap2* aligner (v2.15) implemented in *pbmm2* (v1.9.0) and per-base coverage was tabulated using *mosdepth* (v0.3.3):

```
pbmm2 align --log-level INFO --log-file <OUTPUT_LOG> \
    --preset HiFi --sort --num-threads <THREADS> \
    --sample <SAMPLE_NAME > \
    hs37d5.fa \
    <UNALIGNED_BAM> \
    <OUTPUT_BAM>

mosdepth --threads <THREADS> --use-median \
    --by GRCh37_notinalllowmapandsegdupregions.bed.gz \
    <OUTPUT_PREFIX> <ALIGNED_BAM>
```

Given low depth of coverage, we naively called SNVs within regions defined in the GIAB benchmark BED files supported by ≥2 reads and with minimum mapping quality of 15 using *samtools mpileup* (v1.15.1) and a custom script.

```
samtools mpileup --no-BAQ --fasta-ref hs37d5.fa \
    --positions HG002-4.calling_regions.bed.gz \
    <ALIGNED_BAM> | bgzip > <OUTPUT_PLP_GZ>
```

```
zcat <OUTPUT_PLP_GZ> | plp2vcf.py -q <MIN_MAP_Q> -d <MIN_DEPTH> - | \
      bgzip > <OUTPUT_VCF>
```

For each of HG002, HG003, and HG004, naive SNV calls were intersected with private benchmark SNVs in regions labeled ‘not difficult’ in the GIAB v3.0 genome stratification and covered by ≥2 SMRT-Tag reads using *bedtools* (v2.30.0), *samtools* (v1.15.1), and *bcftools* (v1.15.1). For example, HG002 SMRT-Tag calls were intersected with HG003 benchmark private SNVs as follows:

```
zcat HG002/mosdepth/HG002.per-base.bed.gz | \
awk -v D=2 '{if ($4 >= D) print}' | \
bedtools merge -i - | \
bedtools intersect \
      -u -a - -b GRCh37_notinalldifficultregions.bed.gz | bgzip \
> HG002.d2.GRCh37_notinalldifficultregions.bed.gz

bcftools isec \
      --threads <THREADS> -n =2 -w 1 -c some \
      --regions-file HG002.d2.GRCh37_notinalldifficultregions.bed.gz \
      -Oz -o HG002.q15.d2_vs_HG003_unique.vcf.gz \
      HG002.q15.d2.vcf.gz unique.HG003_GRCh37_1_22_v4.2.1_benchmark.vcf.gz

bcftools index -t -f --threads <THREADS> HG002.q15.d2_vs_HG003_unique.vcf.gz

bcftools stats --threads <THREADS> \
      HG002.q15.d2_vs_HG003_unique.vcf.gz > HG002.q15.d2_vs_HG003_unique.stats

# Determine total number of covered SNVs:
bcftools view --threads <THREADS> \
      --regions-file HG002.d2.GRCh37_notinalldifficultregions.bed.gz \
      -Oz -o HG002.d2_vs_HG003.base.vcf.gz \
      --types snps unique.HG003_GRCh37_1_22_v4.2.1_benchmark.vcf.gz

bcftools index -t -f --threads <THREADS> HG002.d2_vs_HG003.base.vcf.gz

bcftools stats --threads <THREADS> \
      HG002.d2_vs_HG003.base.vcf.gz > HG002.d2_vs_HG003.base.stats
```

**HG002 small variant (SNV and indel) calling and benchmarking**

In addition to the hs37d5 GRCh37 reference genome, GIAB v4.2.1 benchmark VCF and BED files for HG002, and GIAB GRCh37 v3.0 genome stratifications used in the genotype demultiplexing analysis, we downloaded publicly available HG002 PacBio HiFi reads, which were generated by GIAB with ~11 kb size selection and Sequel II chemistry 0.9 and SMRTLink 6.1 pre-release, and are available aligned to the same reference genome:

```
ftp://ftp-
trace.ncbi.nlm.nih.gov/giab/ftp/data/AshkenazimTrio/HG002_NA24385_son/PacBio_SequelII_CCS_11k
b/HG002.SequelII.pbmm2.hs37d5.whatshap.haplotag.RTG.10x.trio.bam
```

We used *pbmm2* for alignment of HG002 SMRT-Tag CCS reads to hs37d5 as before. Median coverage for SMRT-Tag and GIAB PacBio reads was determined using *mosdepth*. CCS reads were subsampled to 3-, 5-, 10-, and 15-fold depths using *samtools* (v1.15.1) based on *mosdepth* median coverage:

```
samtools view --threads <THREADS> --subsample <FRAC>\
    --subsample-seed 0 --bam --with-header --write-index \
    --output <OUTPUT_BAM> \
    <ALIGNED_BAM>
```

Small variants (SNVs and indels) were called using *DeepVariant* (v1.4.0):

```
run_deepvariant --model_type PACBIO --num_shards <THREADS>
    --verbosity 0 --logging_dir <DIR> \
    --reads <ALIGNED_BAM> --ref hs37d5.fa \
    --output_vcf <OUTPUT_VCF>
```

We then compared variants called from SMRT-Tag and GIAB PacBio data against GIAB/NIST v4.2.1 benchmarks[2] using *hap.py* (v0.3.12) and GIAB v3.0 GRCh37 genome stratifications:

```
hap.py -r hs37d5.fa -o <OUTPUT_PREFIX> \
    -f HG002_GRCh37_1_22_v4.2.1_benchmark_noinconsistent.bed \
    --threads <THREADS> --pass-only \
    --engine=vcfeval --verbose --logfile <OUTPUT_LOG> \
    --stratification v3.0-GRCh37-v4.2.1-stratifications.tsv \
    HG002_GRCh37_1_22_v4.2.1_benchmark.vcf.gz \
    <DEEPVARIANT_VCF>
```

**Structural variant calling and benchmarking**

HG002 SMRT-Tag and GIAB Sequel II data were pre-processed as described above for small variant detection. Benchmark NIST Tier 1 SV calls for HG002 (v0.6) and tandem repeats for hg19/hs37d5 were obtained from:

```
https://ftp-
trace.ncbi.nlm.nih.gov/ReferenceSamples/giab/release/AshkenazimTrio/HG002_NA24385_son/NIST_SV_v0
.6/HG002_SVs_Tier1_v0.6.bed
https://ftp-
trace.ncbi.nlm.nih.gov/ReferenceSamples/giab/release/AshkenazimTrio/HG002_NA24385_son/NIST_SV_v0
.6/HG002_SVs_Tier1_v0.6.vcf.gz
ftp://hgdownload.soe.ucsc.edu/goldenPath/hg19/bigZips/hg19.trf.bed.gz
```

Reads were subsampled as described above for small variant analysis. SVs were called using *pbsv* (v2.8.0; https://github.com/PacificBiosciences/pbsv):

```
pbsv discover --hifi --log-level INFO --log-file <LOG_NAME> \
    --tandem-repeats hg19.trf.bed.gz \
    <ALIGNED_BAM> <OUTPUT_PREFIX>.svsig.gz

pbsv call --hifi --log-level INFO --log-file <LOG_NAME> \
    --num-threads <THREADS> \
    hs37d5.fa <OUTPUT_PREFIX>.svsig.gz <OUTPUT_PREFIX>.vcf
```

VCF files output by *pbsv* were compressed and indexed using *samtools*. We then benchmarked variants against the NIST v0.6 Tier 1 SV calls for HG002 using *Truvari*[2] (v3.3.0):

```
truvari bench --comp <PBSV_VCF_GZ> \
    --base HG002_SVs_Tier1_v0.6.vcf.gz \
    --includebed HG002_SVs_Tier1_v0.6.bed \
    --reference hs37d5.fa \
    --output <OUTPUT_PREFIX> / \
    --giabreport --refdist 1000 --pctsim 0 --passonly --debug
```

**Source data for HG002 bisulfite methylation calls**

CpG methylation calls determined using bisulfite sequencing were previously generated as an ONT Benchmark Dataset and were downloaded from:

```
https://ont-open-
data.s3.amazonaws.com/gm24385_mod_2021.09/bisulphite/cpg/CpG.gz.bismark.zero.cov.gz
```

## SUPPLEMENTARY NOTE 1

### Experimental design considerations for PacBio sequencing

The PacBio single-molecule sequencing (SMS) platform is fundamentally different from the Illumina and Oxford Nanopore instruments. There are several technical considerations particular to PacBio SMS that motivated our experimental design for developing and optimizing SMRT-Tag and SAMOSA-Tag. Leveraging the potential of PacBio sequencing (namely, direct detection of DNA modifications), requires library generation without PCR. This leads to a critical limitation, as DNA is lost at every step of library preparation. Importantly, this includes steps required for loading the PacBio sequencer—specifically, polymerase binding and loading on flow cells (SMRTCells). PacBio SMS performance is influenced by several properties: library fragment length distribution, presence of DNA damage, batch-to-batch SMRTCell and polymerase characteristics, and perhaps most importantly, the on-plate loading concentration (OPLC) of libraries. Maximizing the P1 productivity (fraction of zero-mode waveguides sequencing one and only one molecule) and CCS yield (and thus, minimizing cost-per-base) of a PacBio flow cell requires a high per-run OPLC. The only ways to maximize OPLC are by (i) minimizing DNA loss during clean-up steps and (ii) pooling barcoded libraries when possible. We provide salient technical details including OPLC for all SMRT-Tag and SAMOSA-Tag libraries sequenced in this study (**Supplementary Table 3**). While achieving high OPLC to minimize cost-per-base was the primary focus of most experiments presented in this paper, we include as a valuable reference point an experiment where a single library from 40 ng of human gDNA was tagmented and sequenced on a single SMRTCell (**Fig. 2**). This serves to illustrate the capability of SMRT-Tag for maximizing coverage of low-input samples.

### Comparison of input DNA requirements for SMRT-Tag, SAMOSA-Tag, and other methods

SMRT-Tag and SAMOSA-Tag input reduction relative to other methods was estimated based on the following:

- The standard ligation-based PacBio Template Prep Kit 2.0 recommends minimum input of 5 µg DNA, whereas the SMRTbell Prep Kit 3.0 (released in mid-2022) recommends 1 – 5 µg (~170,000 – 800,000 human cells). Taking 40 ng (~7,000 human cells) as a conservative lower bound for SMRT-Tag, the input required relative to these ligation-based methods is 0.8 – 4%, representing reduction of 96 - 99.2%.

- The input amounts reported in the publications describing single-molecule chromatin profiling methods are: SAMOSA[3,4] / Fiber-seq[5] (2 µg), DiMeLo-seq[6] (6 – 30 µg), SMAC-seq[7] (6 µg), nanoNOMe[8] (2 – 3 µg), and MeSMLR-seq[9] (quantity not reported, but minimum quoted for the ONT Ligation Sequencing Kit is 1 µg). SAMOSA-Tag experiments used 30,000 – 50,000 nuclei (~180-300 ng DNA). Noting that direct comparison is challenging given that the substrate for SAMOSA-Tag is chromatin and not purified DNA, the input needed relative to other chromatin profiling methods is 0.6 – 9%, representing reduction of 91 – 99.4%.

Accordingly, we conservatively estimate that SMRT-Tag requires 1 – 5% as much DNA as ligation-based library preparation (equating to reduction by 95 – 99%) and SAMOSA-Tag requires 1 – 10% of the input reported for comparable methods (corresponding to reduction by 90 – 99%). Therefore, SMRT-Tag and SAMOSA-Tag reduce the magnitude of input required by approximately 1 or 2 orders (*i.e.*, 10-fold or 100-fold).

## SUPPLEMENTARY NOTE 2

Tagmentation-based library preparation and SMRT-Tag and SAMOSA-Tag are flexible. We highlight technical considerations for optimizing these methods and alternative approaches to suit an investigator's use case.

### Tradeoff between molecule length and molarity

In preparing a PacBio library of a given mass, the number of molecules is inversely proportional to the fragment length. Given mass *m* in nanograms and length *L*, the number of picomoles of DNA can be estimated as, *e.g.*, $m \times 10^3 / (660 \times L)$ where 660 pg/pmol is the average molecular weight of a base pair. Therefore, tagmenting gDNA into very long fragments may yield a library below the on plate loading concentration (OPLC) lower bound of 20 – 40 pM (*i.e.*, 2.3 – 4.6 fmol in a 115 µL volume) for Sequel II SMRTCells. On the other hand, if input DNA is not limiting, it may be reasonable to target longer fragments. Based on the mean library conversion efficiency of ~20% (**Supplementary Table 3**) and the relationship between mass and length of DNA, the input required for a particular library size can be readily estimated. For example, to achieve an OPLC of 37 pM (volume: 115 µL) for libraries with median lengths of 2.3, 10, and 100 kb, the starting material required is approximately 35, 150, and 1,500 ng, respectively. Considerations related to length and molar quantity are not unique to PacBio sequencing. For example, multiplexing of low-input (50 – 100 ng) libraries prepared using the Tn5-based Oxford Nanopore Rapid sequencing kit (Cat. No. SQK-RAD114) is often required to reduce per-sample cost.

### Input DNA quality

PacBio's sequencing-by-synthesis chemistry relies on processive polymerization on a native, circular template. High-quality, high molecular weight (HMW) DNA is therefore required for PacBio HiFi or circular consensus sequencing (CCS). There are several approaches for assessing input quality. Automated (*e.g.*, Agilent Femto Pulse) or manual (*e.g.*, BioRad CHEF-DR II) pulsed field gel electrophoresis systems are the gold-standard but can be cumbersome. Alternatively, 10 – 25 ng DNA loaded on a 0.4 – 0.6% TAE/agarose gel run at low voltage (60-80V) for 2-3 h and stained with SYBR gold for 15 min can provide an estimate of sample degradation, which would appear as a smear <10 kb. Finally, gDNA ScreenTape (Agilent) can be used to assess DNA quality, though results can be variable. For reference, control gDNA used in this study without damage repair (as is standard in PacBio TPK2.0) had a DNA integrity number (DIN) of 9.7. In our hands, samples that were degraded and did not yield libraries had DIN <9.2. DNA can be purified using standard approaches such as phenol:chloroform:isoamyl alcohol extraction or commercially available kits (*e.g.*, Promega Wizard, New England Biolabs Monarch, and Qiagen MagAttract), which all produced gDNA with DIN >9.5 that could be successfully converted to SMRT-Tag libraries in our hands. Based on our experience, we suggest targeting DIN ≥9.5.

### Tagmentation conditions

*Determining conditions for an application of interest*

The key parameter for Tn5-based PacBio library preparation is transposome concentration, which must be determined empirically for a given batch of Tn5 complexed with hairpin adaptors and for a given application. Note that input DNA mass and quality are also important considerations, but these may be constrained to a degree by, *e.g.*, the amount of material available. Pilot experiments using a dilution series of transposome and/or input DNA obtained from a source comparable to the intended application are critical for optimizing tagmentation. We suggest analyzing libraries obtained from pilot studies via gel electrophoresis or on an instrument such as TapeStation, BioAnalyzer, or Femto Pulse (Agilent). Multiplexing and sequencing libraries at low depth (*e.g.*, **Supplementary Fig. 4**) can confirm that molecules in the desired length range are captured. The effect of transposome concentration, input DNA mass, and reaction temperature are discussed below.

*Transposome concentration*

Loading of Tn5 transposomes onto DNA can be approximated as a Poisson process (*i.e.*, the number of Tn5 complexes per DNA fragment varies according to the amount of Tn5), and the exact position of each complex on single molecules is essentially random. The size of the resulting fragments, which represent the interstitial region between adjacent transposition sites, is thus the difference between adjacent realizations of a uniform random variable U(1, *molecule length*) and can be approximated by an exponential distribution. Therefore, under

concentrations used for tagmentation, Tn5 has a tendency to generate short fragments. The triple-mutant Tn5 enzyme used here permits transposome concentration-dependent control of fragment lengths[10], which we confirmed initially based on analytical gel electrophoresis of tagmented gDNA (**Fig. 1b**). To better characterize the relationship between transposome concentration and fragment length, we performed SMRT-Tag on inputs ranging 40 – 1,000 ng and Tn5 monomer amounts of 0.005 – 5 pmol (at least two orders of magnitude for each parameter; **Supplementary Fig. 4**). Libraries were multiplexed and sequenced to low coverage, confirming the inverse relationship between Tn5 and DNA amounts on resulting fragment lengths. For example, 200 ng gDNA tagmented with the equivalent of 0.05 pmol Tn5 monomer at 55ºC generated libraries of mean length ~3-5 kb, whereas the same amount of DNA tagmented with 5 pmol Tn5 at 55ºC yielded molecules with ~500 bp average length (**Supplementary Fig. 4**, **Supplementary Table 3**).

Given these observations, we propose a simple procedure for calibrating the amount of hairpin-loaded Tn5 to generate a library of a specific mean size: First, using a fixed amount of gDNA (such as the 160 ng experiments in this study, **Supplementary Table 3**), carry out tagmentation with a dilution series (*e.g.*, 1:16, 1:64, 1:128, etc.) of hairpin-loaded Tn5 stock (9.4µM monomer) coupled with analytical electrophoresis or shallow multiplex sequencing to estimate the relationship between Tn5 quantity and library size distribution. Then, for a target library size (*e.g.*, 3 – 5 kb), the amount of Tn5 can be normalized per mass gDNA (*n* pmol Tn5 / *m* ng gDNA) to produce a ratio that is, in our hands, approximately scalable to a range of input quantities. As an example, for the transposomes assembled for this study, experiments using 160 ng of gDNA suggested that Tn5 monomer range from 0.073 – 0.146 pmol could consistently generate libraries with mean lengths of 2 – 5 kb. This yielded Tn5 monomer:gDNA ratios of $4.6 \times 10^{-4}$ – $9.3 \times 10^{-4}$ (pmol:ng). Scaled to 40 ng gDNA, this gave a Tn5 amount of 0.018 – 0.037 pmol, which generated the expected library distributions of 2 – 5 kb (**Supplementary Fig. 4b**). This relationship held across the batches of barcoded hairpin-loaded Tn5 that were prepared in this study. This calibration procedure should be repeated for every batch of transposome to ensure reproducibility. Further, based on the particulars of the input material and assay, pilot experiments titrating different reaction conditions are the best way to guide parameter selection. For example, the amount of transposome required for *in situ* SAMOSA-Tag (wherein the transposition reaction occurs in intact nuclei) was much higher and determined based on reported concentrations used for ATAC-seq.

*Input DNA mass*

Tagmentation has a wide theoretical input range with lower bound on the picogram scale (*i.e.*, single cells). Taking into consideration the mass/molar quantity tradeoff and minimum OPLC of 20 – 40 pM for PacBio sequencing, the lowest amount of gDNA used for library preparation in this study was 40 ng. In experiments performed to guide parameter selection (**Supplementary Fig. 4**), we tagmented up to 1,000 ng of DNA. At the typical volumes used for library preparation (<10 µL), HMW gDNA can be quite viscous and challenging to handle. Though future modifications may enable use of large input amounts, we consider ~250 ng to be a soft upper limit for tagmentation-based library preparation. Input DNA quality (see above) is an additional consideration that may affect the mass required for conversion to library molecules – *i.e.*, for a low-quality sample, more input material would be required to generate sufficient sequenceable templates after exonuclease digestion.

*Reaction temperature*

Tagmentation is typically performed at 55ºC, the temperature optimal for Tn5 activity. However, Tn5 retains activity at lower temperatures. Both the conventionally used double-mutant and/or the triple-mutant enzymes used here have been shown in this study (**Fig. 1b, Supplementary Fig. 4**) and others[11] to favor generation of longer fragments at 37ºC. Note that in contrast to the gel-based analysis of tagmented DNA in **Fig. 1b**, libraries generated under a variety of reaction conditions were multiplexed for sequencing in the analysis presented in **Supplementary Fig. 4**. Wide variation in length between libraries affected estimation of loading and sequencing characteristics, which may have obscured some temperature-dependent differences. In our hands, carrying out tagmentation at 55ºC was sufficient for generating libraries of mean lengths in the 1-7 kb range (**Supplementary Table 3**); however, in applications targeting much longer fragment lengths, it may be reasonable to lower the reaction temperature to ≥37ºC. For example, several ATAC-seq protocols use a lower temperature for tagmentation (37ºC) to better preserve native chromatin structure.

*Other considerations*
In this study, we did not directly test the effect of crowding agents (*e.g.*, polyethylene glycol) on tagmentation efficiency and library characteristics. However, prior work suggests that modulating the type and concentration of crowding agents may help tune input quantity and library size[12].

**Size selection**
Bead-based cleanup can be optionally performed to shift the distribution of fragment sizes in the library at the cost of losing a portion of molecules. Though SMRT-Tag and SAMOSA-Tag libraries can generally be sequenced without size selection using polymerase 2.1/3.1 (see below), given that Tn5 tagmentation is a Poisson process, there can be a preponderance of short (<700bp) fragments. These may be overlooked in fluorescence-based quantification assays despite constituting a significant fraction of the library. In cases where high concentrations of Tn5 are used or where quality control analyses suggest a large population of short fragments, depleting these molecules can improve loading efficiency by aligning the library length distribution to the preference of PacBio polymerases 2.1/3.1 vs 2.2/3.2. In our hands, depleting <700bp or <3kb fragments reduced the fraction of short reads in libraries sequenced with polymerase 2.2 and permitted more accurate estimation of mean fragment length during the sequencing loading reaction. 'Double-sided' cleanup wherein short and long fragments are sequenced separately is adapted from an older version of PacBio's Iso-Seq protocol in which short fragments depleted from the library are recovered and sequenced to maximize use of input DNA. This is not required for SMRT-Tag or SAMOSA-Tag but may be a consideration if starting material is limiting.

**Choice of PacBio polymerase**
Per manufacturer recommendations, libraries with mean fragment length <3 kb should be sequenced with polymerase 2.1/3.1, whereas polymerases 2.2/3.2 are better suited for libraries with mean fragment length >3 kb. This is based in part on general characteristics of the enzymes/sequencing chemistry – *i.e.*, 2.2 / 3.2 polymerase is highly processive and produces longer reads but is, in our hands, typically less tolerant to poor estimation of mean library size during loading. In general, we find that libraries with mean lengths as high as ~6 kb can be adequately sequenced with polymerase 2.1. Empirically, we recommend taking into consideration the relative fraction of molecules in a library with length <3kb in choosing which polymerase to use given that some deviation from the manufacturer recommended cutoff is tolerated.

***In situ* vs. *ex situ* SAMOSA-Tag**
We describe both *in situ* (tagmentation occurs following EcoGII methylation in intact nuclei) and *ex situ* (DNA is purified from EcoGII methylated nuclei and then subjected to tagmentation) versions of SAMOSA-Tag. *Ex situ* SAMOSA-Tag is essentially SMRT-Tag carried out using SAMOSA DNA as input, highlighting the flexibility of Tn5-based library preparation. Depending on the anticipated application, one approach may be preferred over the other. *In situ* tagmentation has the benefit of avoiding DNA extraction and attendant losses and preferentially samples open chromatin regions evinced by transposition adjacent to barrier elements (**Fig. 3c**) producing ATAC-seq-like coverage profile (**Supplementary Fig. 19**). This could be ideal in input- and sequencing depth-limited settings where the primary biological interest is, *e.g.*, gene regulatory regions. On the other hand, *ex situ* SAMOSA-Tag delivers more uniform coverage as suggested by abrogation of the barrier effect (**Fig. 3c**) and may be better suited for studies requiring even genome sampling such as analysis of heterochromatic regions and integrated whole genome assembly and epigenome profiling.

**Alternative approaches**
Applications such as variant discovery, *de novo* assembly, and epigenome profiling in heterogenous samples may require high depth of sequencing coverage and/or sampling of sufficiently long molecules. Given the impact on coverage of the fragment length/molarity tradeoff (see above) and that tagmentation conditions explored here generate median lengths short of the 15-20 kb capability of PacBio SMS, methods that use ligation (*e.g.* conventional SAMOSA[3,4]) or ONT (*e.g.*, NanoNOMe[8]) may be considered if input material is not limiting.

## SUPPLEMENTARY REFERENCES


1. Zook, J. M. *et al.* Extensive sequencing of seven human genomes to characterize benchmark reference materials. *Scientific data* vol. 3 160025 (2016).
2. English, A. C., Menon, V. K., Gibbs, R. A., Metcalf, G. A. & Sedlazeck, F. J. Truvari: refined structural variant comparison preserves allelic diversity. *Genome Biol.* **23**, 271 (2022).
3. Abdulhay, N. J. *et al.* Massively multiplex single-molecule oligonucleosome footprinting. *Elife* **9**, (2020).
4. Abdulhay, N. J. *et al.* Nucleosome density shapes kilobase-scale regulation by a mammalian chromatin remodeler. *Nat. Struct. Mol. Biol.* (2023) doi:10.1038/s41594-023-01093-6.
5. Stergachis, A. B., Debo, B. M., Haugen, E., Churchman, L. S. & Stamatoyannopoulos, J. A. Single-molecule regulatory architectures captured by chromatin fiber sequencing. *Science* **368**, 1449–1454 (2020).
6. Altemose, N. *et al.* DiMeLo-seq: a long-read, single-molecule method for mapping protein-DNA interactions genome wide. *Nat. Methods* **19**, 711–723 (2022).
7. Shipony, Z. *et al.* Long-range single-molecule mapping of chromatin accessibility in eukaryotes. *Nat. Methods* **17**, 319–327 (2020).
8. Lee, I. *et al.* Simultaneous profiling of chromatin accessibility and methylation on human cell lines with nanopore sequencing. *Nat. Methods* **17**, 1191–1199 (2020).
9. Wang, Y. *et al.* Single-molecule long-read sequencing reveals the chromatin basis of gene expression. *Genome Res.* **29**, 1329–1342 (2019).
10. Hennig, B. P. *et al.* Large-scale low-cost NGS library preparation using a robust Tn5 purification and tagmentation protocol. *G3: Genes, Genomes, Genetics* **8**, 79–89 (2018).
11. Vonesch, S. C. *et al.* Fast and inexpensive whole-genome sequencing library preparation from intact yeast cells. *G3 (Bethesda)* **11**, 1–12 (2021).
12. Picelli, S. *et al.* Tn5 transposase and tagmentation procedures for massively scaled sequencing projects. *Genome Res.* **24**, 2033–2040 (2014)
13. Yu, H.-B., Johnson, R., Kunarso, G. & Stanton, L. W. Coassembly of REST and its cofactors at sites of gene repression in embryonic stem cells. *Genome Res.* **21**, 1284–1293 (2011).

## SUPPLEMENTARY FIGURES

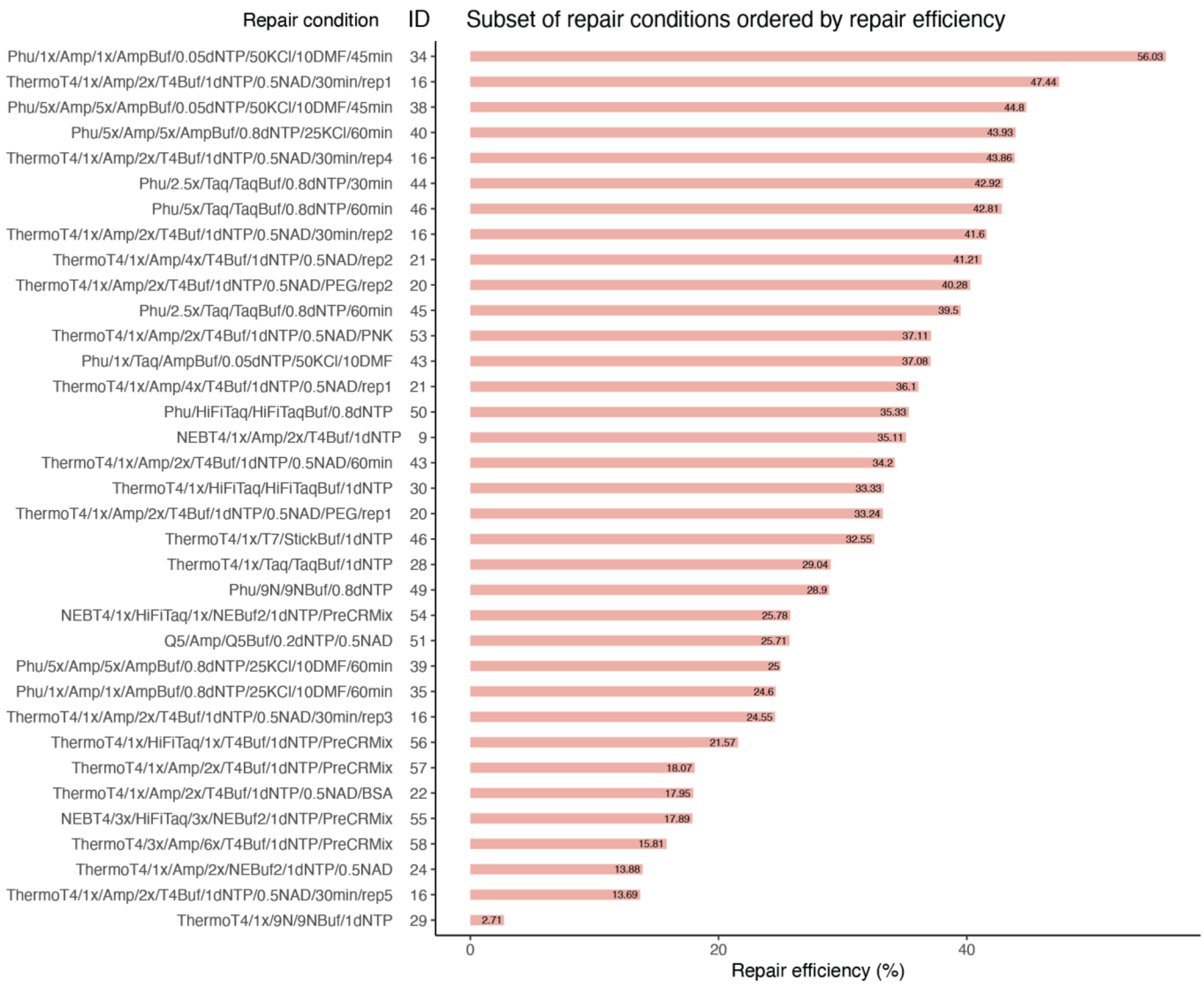


**Supplementary Figure 1: Repair efficiency for a subset of the 62 conditions tested to optimize gap repair.** Repair efficiency (defined as percent yield of product compared to input DNA by mass following exonuclease treatment) for 35 of the 62 conditions tested. A mixture of Phusion polymerase and Taq ligase was selected for gap repair as these provided the most consistently high repair efficiency across multiple experiments.

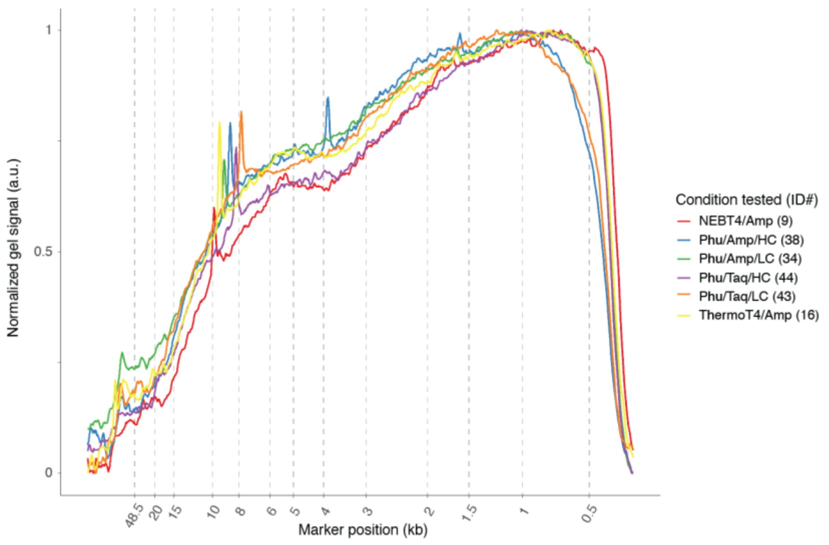


**Supplementary Figure 2: Example analytical gel trace for validating the size distribution of products for a subset of gap repair conditions.** In addition to repair efficiency, we also validated that gap repair conditions did not appreciably change the size distribution of resulting libraries by gel electrophoresis. Shown here are analytical gel traces for six specific conditions tested in this study, including Phusion / Taq in multiple buffers.

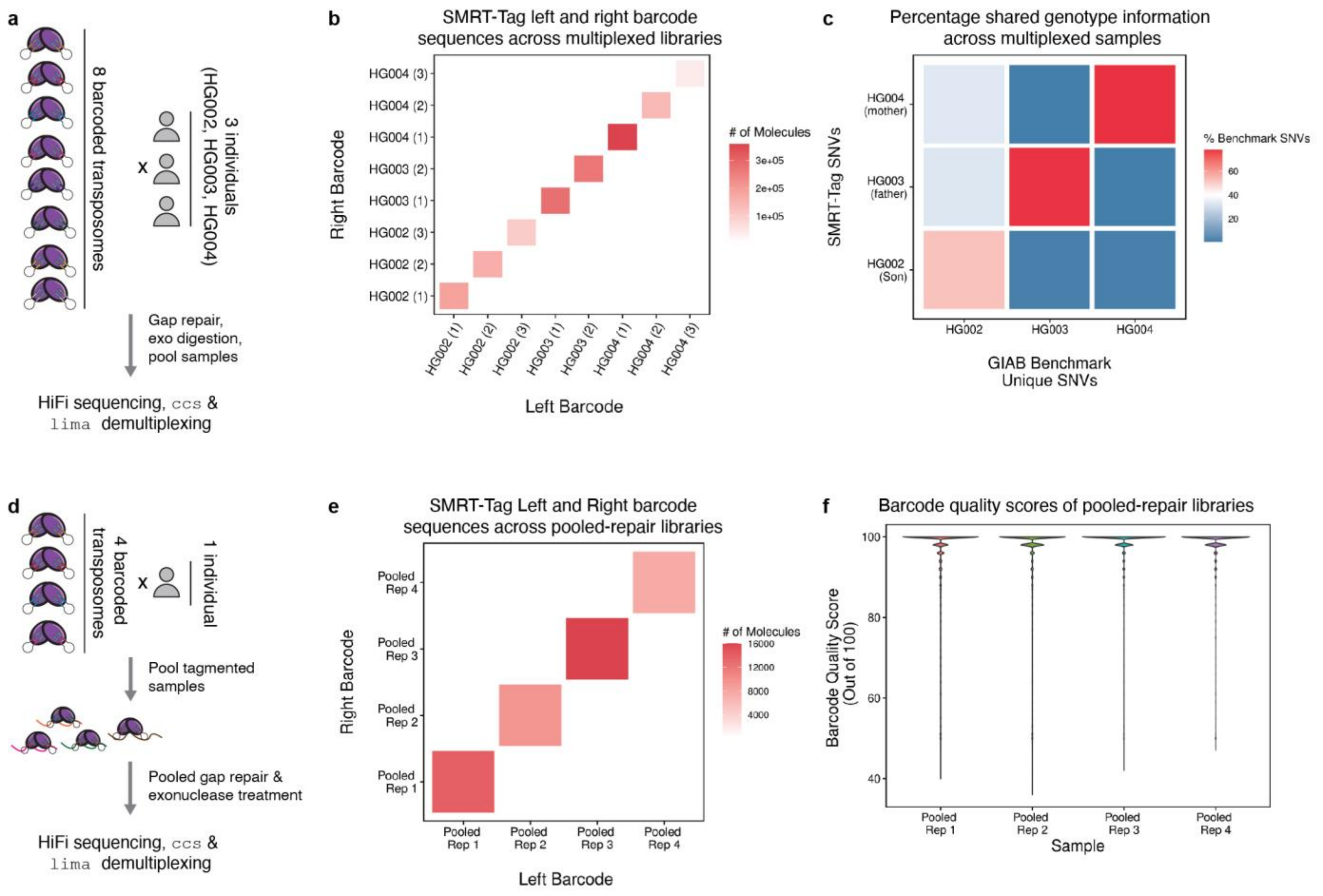


**Supplementary Figure 3: Control experiments to establish multiplexing with SMRT-Tag. (a)** Overview of genotype mixing experiment wherein gDNA from the HG003, HG004, and HG002 human trio were individually barcoded with one of 8 uniquely loaded transposomes, gap-repaired, and exo-treated prior to pooling for sequencing. **(b)** Heatmap of results from PacBio's *lima* demultiplexer, which annotates molecules with matching barcodes, versus those with mixed barcodes. Signal along the diagonal demonstrates minimal cross-contamination between barcoded samples. **(c)** Percentage shared genotype across barcoded samples. HG002 (child) shares SNVs with HG003 and HG004 (parents), but HG003 and HG004 (parents) have minimal genotype overlap. This analysis considered all 'private' SNVs across HG003 and HG004. **(d)** Overview of experiment to validate pooled gap repair without pervasive barcode hopping wherein gDNA from one individual was barcoded with one of four different transposomes prior to pooled gap repair, exo digestion, and sequencing. **(e)** As in **b** but for pooled experiment in **d**. **(f)** Distributions of *lima* quality scores for barcoded molecules from **d**.

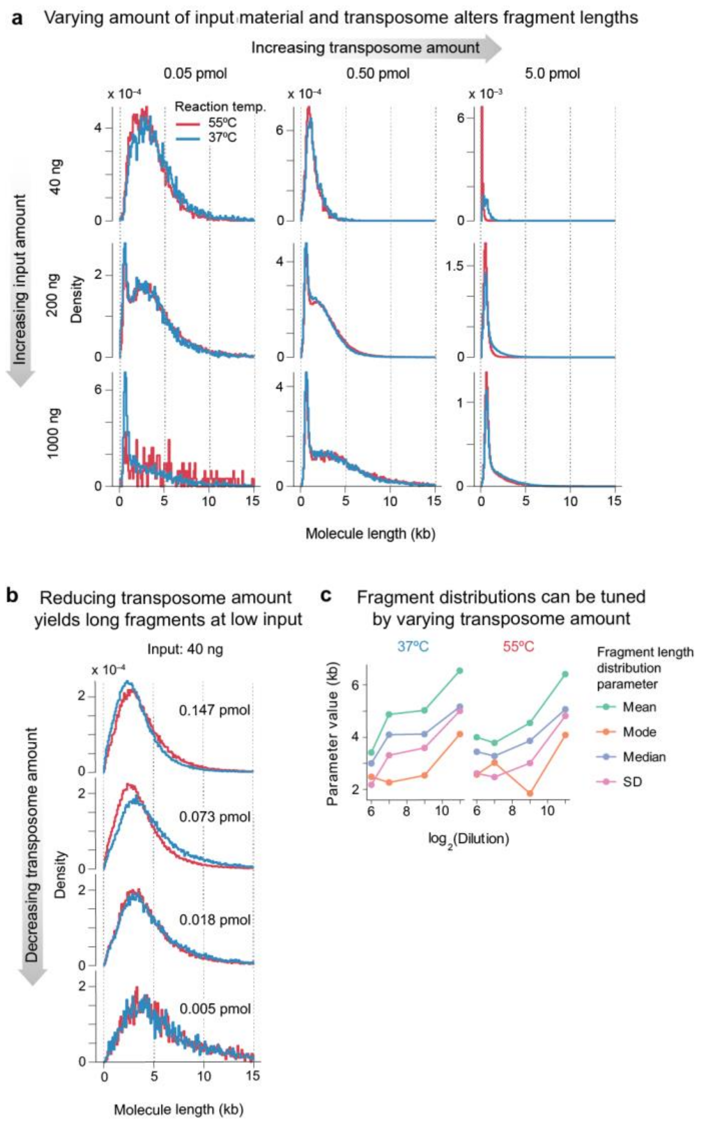


**Supplementary Figure 4: Effect of Tn5 concentration, input amount, and temperature on tagmentation. (a)** CCS fragment length distributions for SMRT-Tag libraries constructed by varying Tn5 concentration (columns) and input amount (rows) at 55ºC (red curves) and 37ºC (blue curves). **(b)** Effect of varying transposome amount keeping input DNA quantity fixed at 40 ng. **(c)** Quantification of mean, mode, median, and standard deviation (S.D.) for each sequenced library as a function of transposome dilution factor.

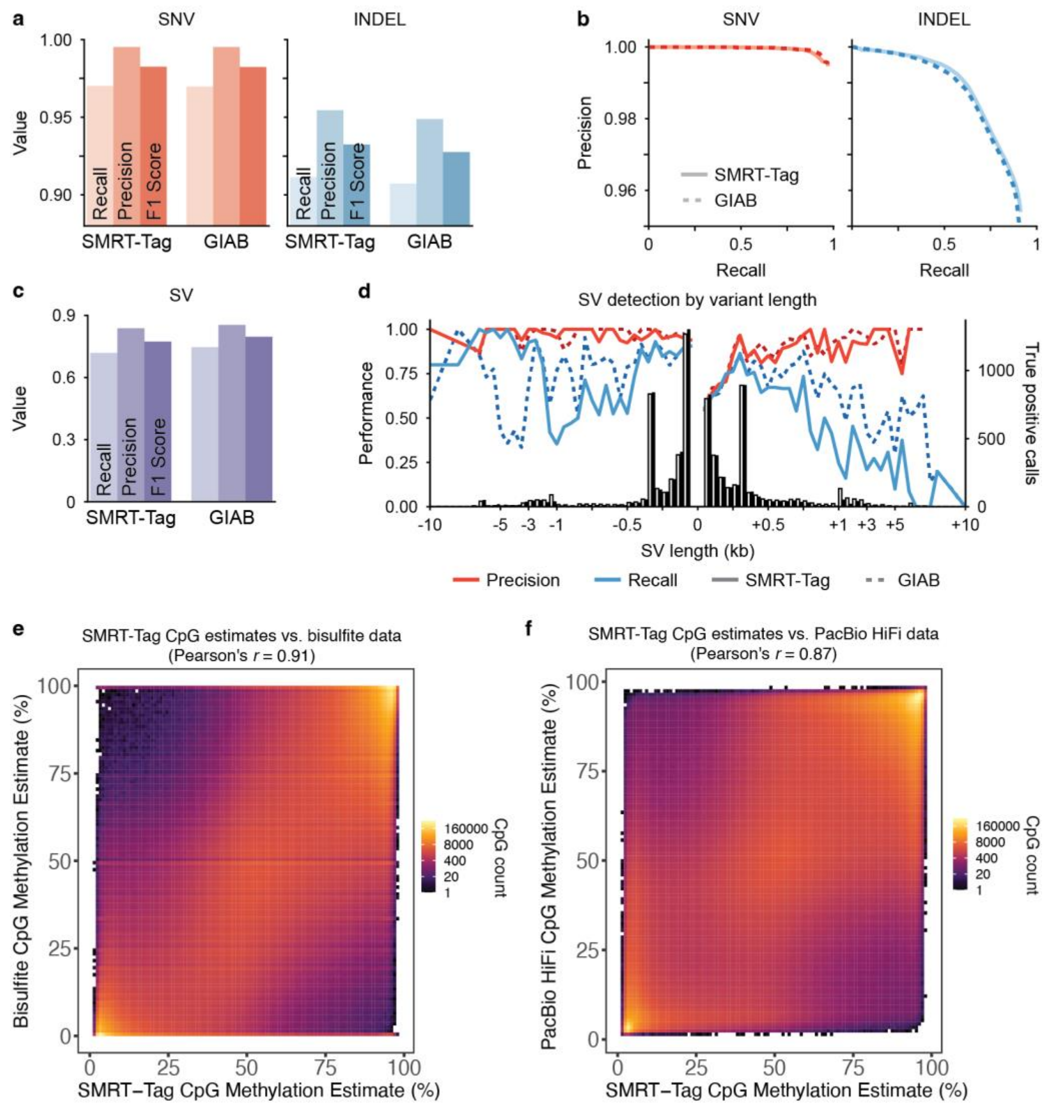


**Supplementary Figure 5: Benchmarking high coverage HG002 SMRT-Tag and ligation-based PacBio libraries against GIAB and CpG methylation standards. (a)** Precision, recall, and F1 scores for *DeepVariant* single nucleotide variant (SNV) and insertion / deletion (indel) calls from high-coverage SMRT-Tag libraries and coverage-matched, ligation-based PacBio data compared against GIAB truth sets. **(b)** Precision as a function of recall for SNVs and indels for SMRT-Tag and ligation-based PacBio data benchmarked against GIAB truth sets. Performance characteristics **(c)** in aggregate and **(d)** binned by structural variant (SV) size for *pbsv* calls from SMRT-Tag and coverage-matched, ligation-based GIAB PacBio data benchmarked against the GIAB SV call set. Comparison of SMRT-Tag *primrose* CpG methylation against **(e)** bisulfite and **(f)** ligation-based PacBio data.

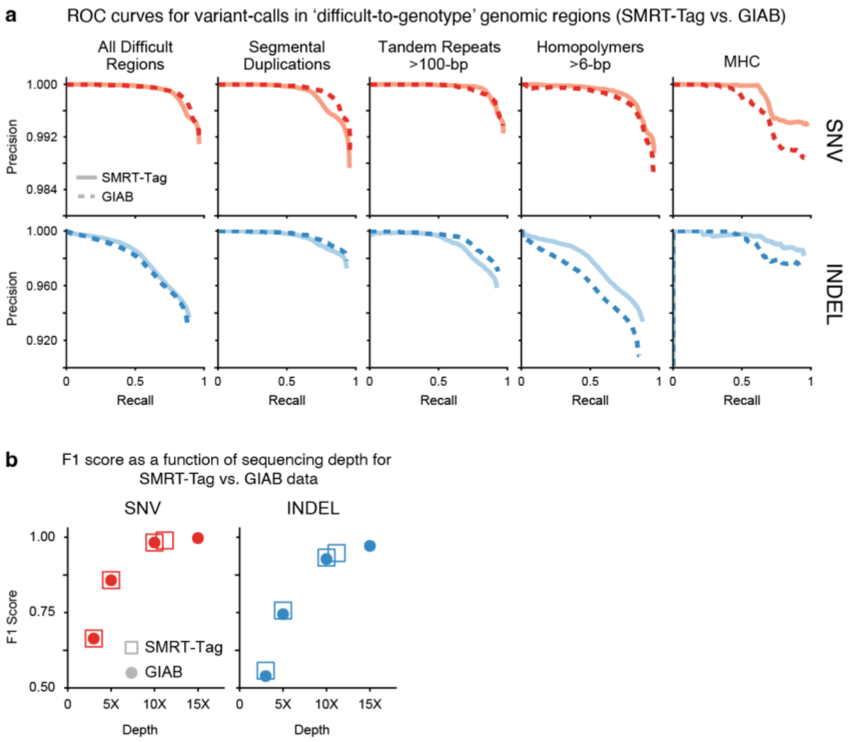


**Supplementary Figure 6: Performance of SMRT-Tag in difficult-to-genotype regions and as a function of sequencing depth. (a)** *DeepVariant* precision-recall curves for SNV (red) and indel (blue) calls in challenging genomic regions, including segmental duplications, tandem repeats, homopolymers, and the MHC locus, for high-coverage SMRT-Tag data (solid) versus coverage-matched, ligation-based GIAB PacBio data (dashed). **(b)** Composite F1 score for SMRT-Tag (closed circles) versus GIAB data (open squares) as a function of sequencing depth, for SNV (red) and indel (blue) calls.

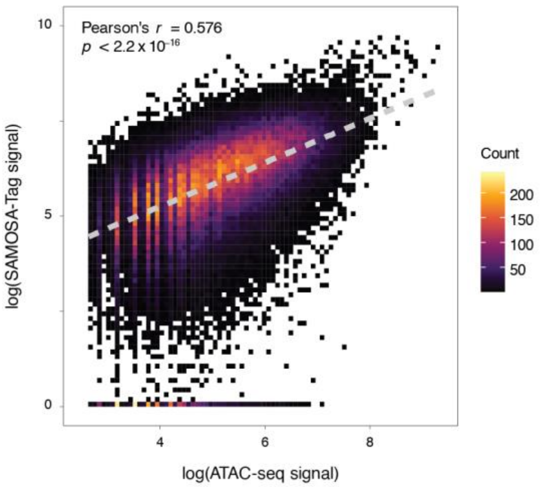


**Supplementary Figure 7: Genome-wide correlation of OS152 SAMOSA-Tag and ATAC-seq accessibility.** SAMOSA-Tag methyltransferase and ATAC-seq transposase accessibility are positively correlated (Pearson's *r* = 0.576, two-sided $p < 2.2 \times 10^{-16}$ as reported by the R v4.2.1 *cor.test* function).

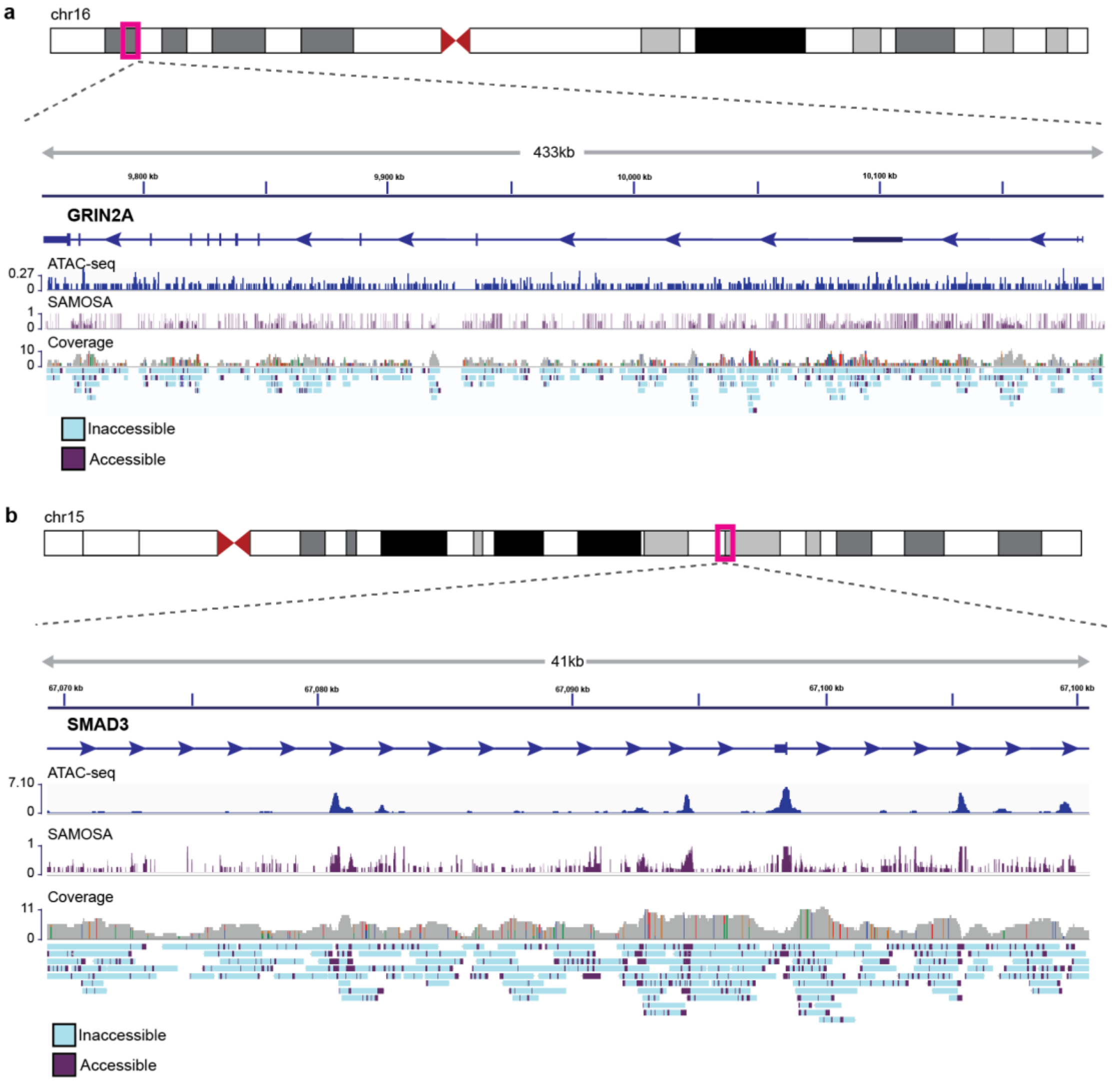


**Supplementary Figure 8: Examples of SAMOSA-Tag coverage and signal plotted with ATAC-seq data for copy-number loss (*GRIN2A*) and copy-number neutral (*SMAD3)* loci.**

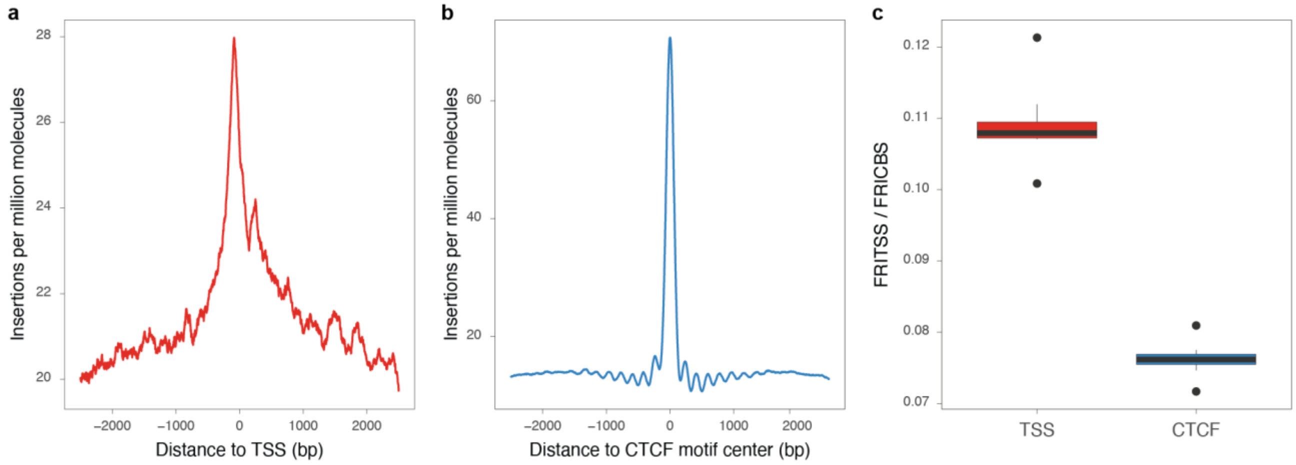


**Supplementary Figure 9: Subtle insertional preference at transcription start sites and CTCF motifs in OS152 SAMOSA-Tag experiments**. Metaplots of insertions per million sequenced OS152 SAMOSA-Tag molecules in 5-kb windows centered at **(a)** hg38 transcription start sites (TSSs) and **(b)** U2OS ChIP-seq-backed CTCF binding sites. Signal was smoothed using a 100-nt running mean. **(c)** Fraction of insertions in TSS (FRITSS; *n* = 44,178) and in CTCF binding sites (FRICBS; *n* = 26,896) across all eight replicate experiments. Boxplot, center = median; upper and lower bounds = interquartile range (IQR); whiskers = 1.5 x IQR.

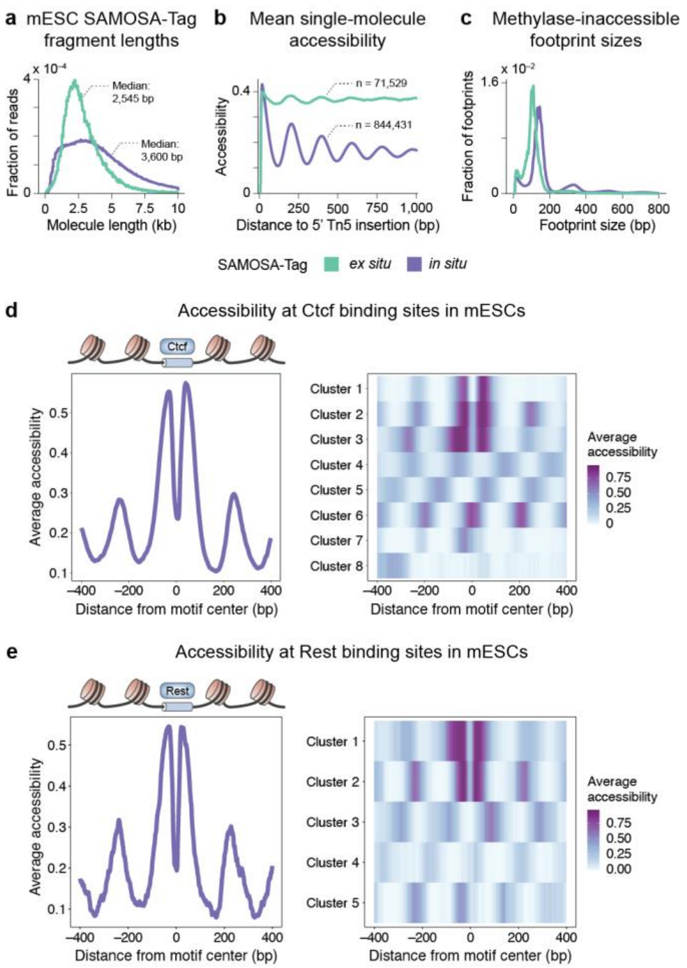


**Supplementary Figure 10: SAMOSA-Tag generalizes to different cell types, can be performed *in situ* or *ex situ*, and can footprint factors other than CTCF/Ctcf. (a)** Fragment length distributions, **(b)** mean single-molecule $m^6dA$ accessibility, and **(c)** sizes of EcoGII methylase-inaccessible footprints in mouse embryonic stem cells (mESCs) for SAMOSA-Tag performed *in situ* (tagmentation of intact nuclei after EcoGII treatment; purple) and *ex situ* (tagmentation of DNA extracted from nuclei after EcoGII treatment; green). **(d)** *In situ* mESC SAMOSA-Tag molecules were clustered into 8 single-molecule accessibility patterns around Ctcf sites predicted using ChIP-seq data. **(e)** As in **d** but for Nrsf / Rest centered at sites predicted using published ChIP-seq data[13].

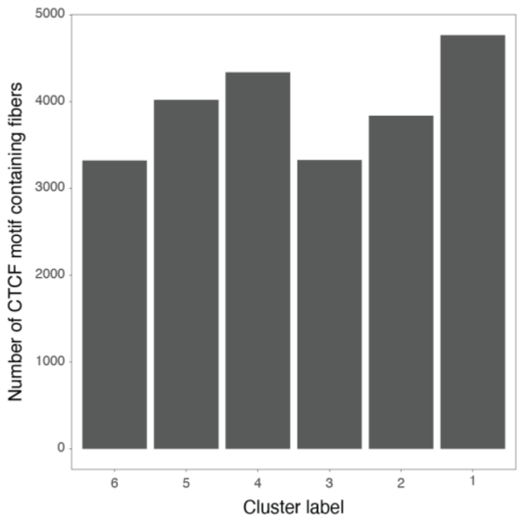


**Supplementary Figure 11: Cluster sizes resulting from Leiden clustering of single-molecule accessibility patterns surrounding predicted CTCF sites.** Cluster labels match **Fig. 4b,c**.

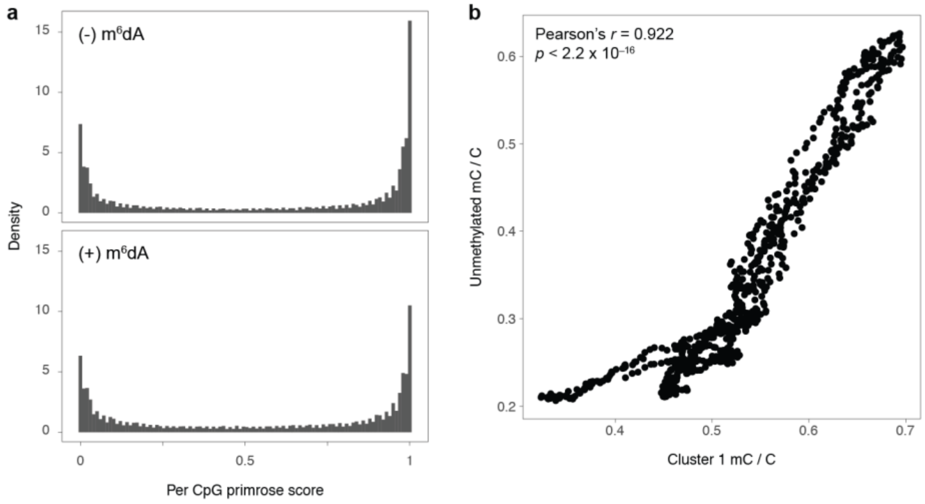


**Supplementary Figure 12: m$^6$dA footprinting does not appreciably impact CpG methylation detection. (a)** Distribution of per-CpG *primrose* scores (50,000 sampled CpGs per experiment) for negative control experiments where EcoGII was omitted (no m$^6$dA; top) and SAMOSA-Tag experiments (bottom). **(b)** Correlation of average CpG methylation from SAMOSA-Tag molecules with detectable m$^6$dA signal (cluster 1, **Fig. 4b,c**) versus without appreciable adenine methylation around predicted CTCF sites (Pearson's $r = 0.922$, two-sided $p < 2.2 \times 10^{-16}$ as reported by the R v4.2.1 *cor.test* function).

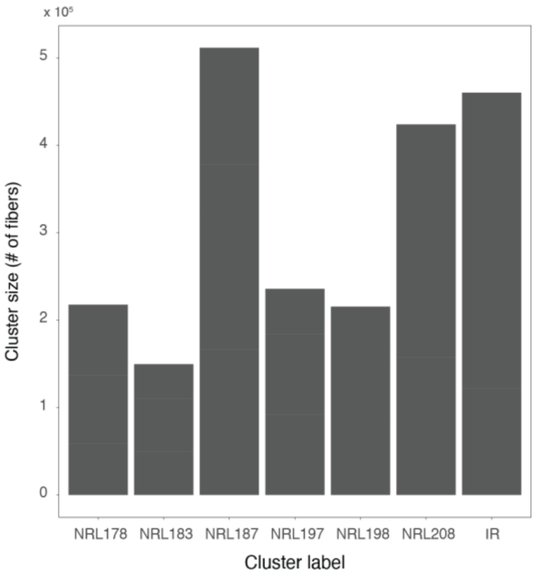


**Supplementary Figure 13: Fiber type cluster sizes resulting from Leiden clustering of SAMOSA-Tag accessibility autocorrelation.** Cluster labels match **Fig. 4e,f**.

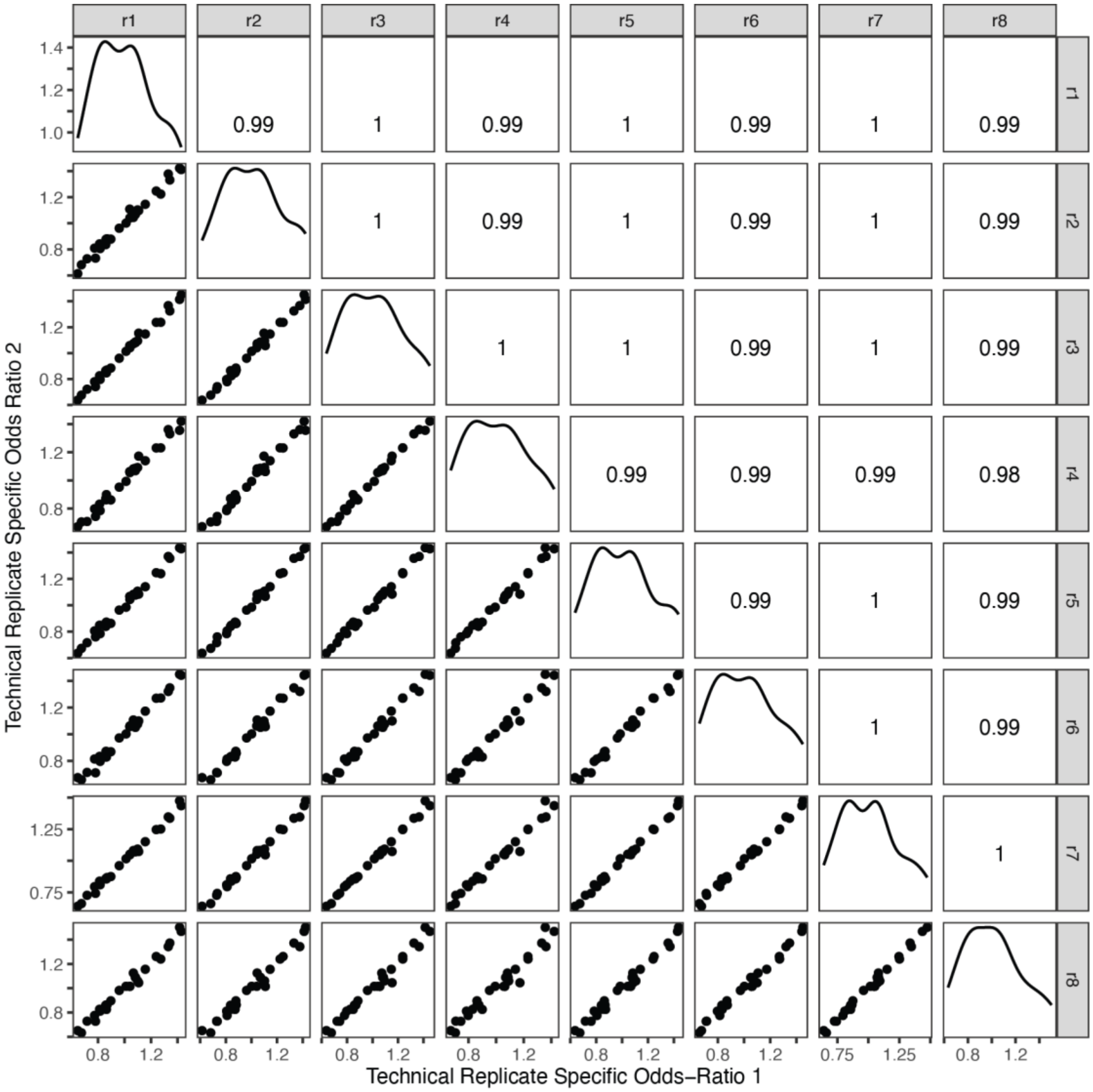


**Supplementary Figure 14: SAMOSA-Tag fiber enrichments in differential CpG content / CpG methylation bins are technically reproducible.** Matrix of scatter plots with Pearson's *r* correlation values across each of eight replicate OS152 SAMOSA-Tag experiments.

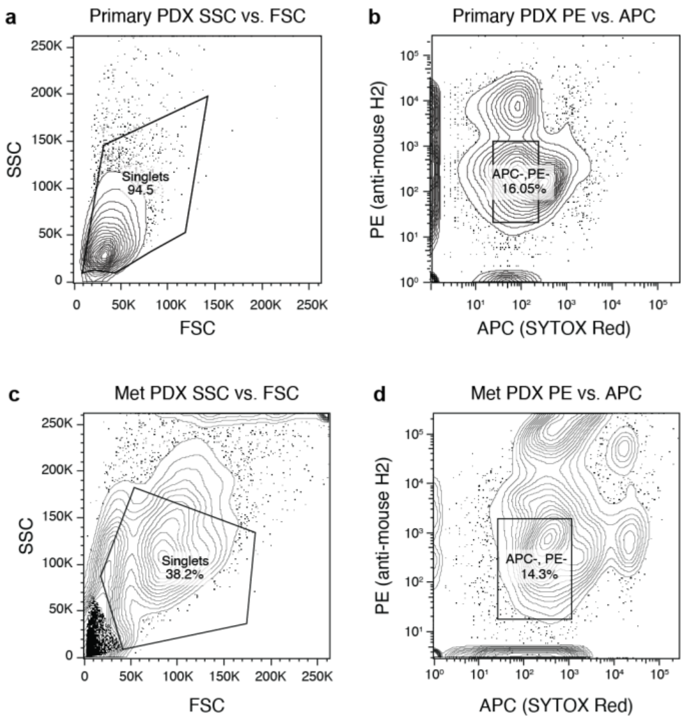


**Supplementary Figure 15: FACS gating strategy for PDX live-dead / human-mouse sorts**. **(a,b)** Primary prostate tumour PDX sorts. **(c,d)** Metastatic prostate tumour PDX sorts.

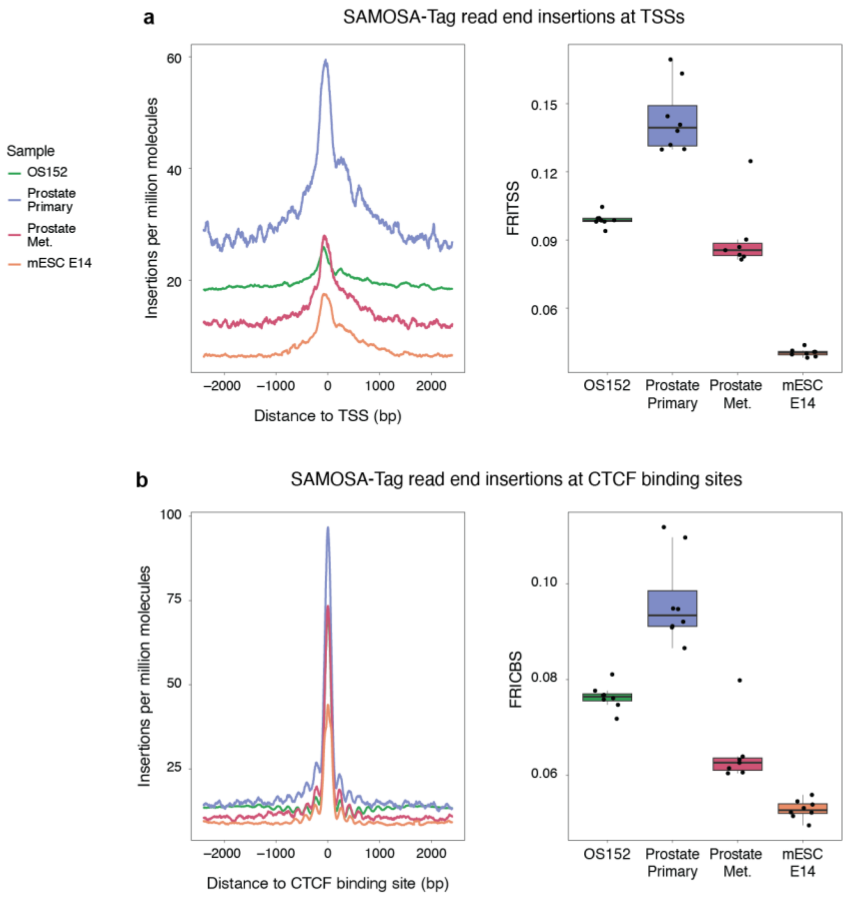


**Supplementary Figure 16: Comparison of insertion preference in PDX and cell line SAMOSA-Tag experiments.** Insertion preference (left) and FRITSS/FRICBS scores (right) at **(a)** TSSs ($n$ = 44,178) and **(b)** ChIP-backed CTCF binding sites ($n$ = 26,896) for technical replicates of cell line (OS152, $n$ = 8 and mESC E14, $n$ = 8) and PDX SAMOSA-Tag (primary, $n$ = 8 and metastasis, $n$ = 7) data. Boxplot: center = median; upper and lower bounds = interquartile range (IQR); whiskers = 1.5 x IQR.

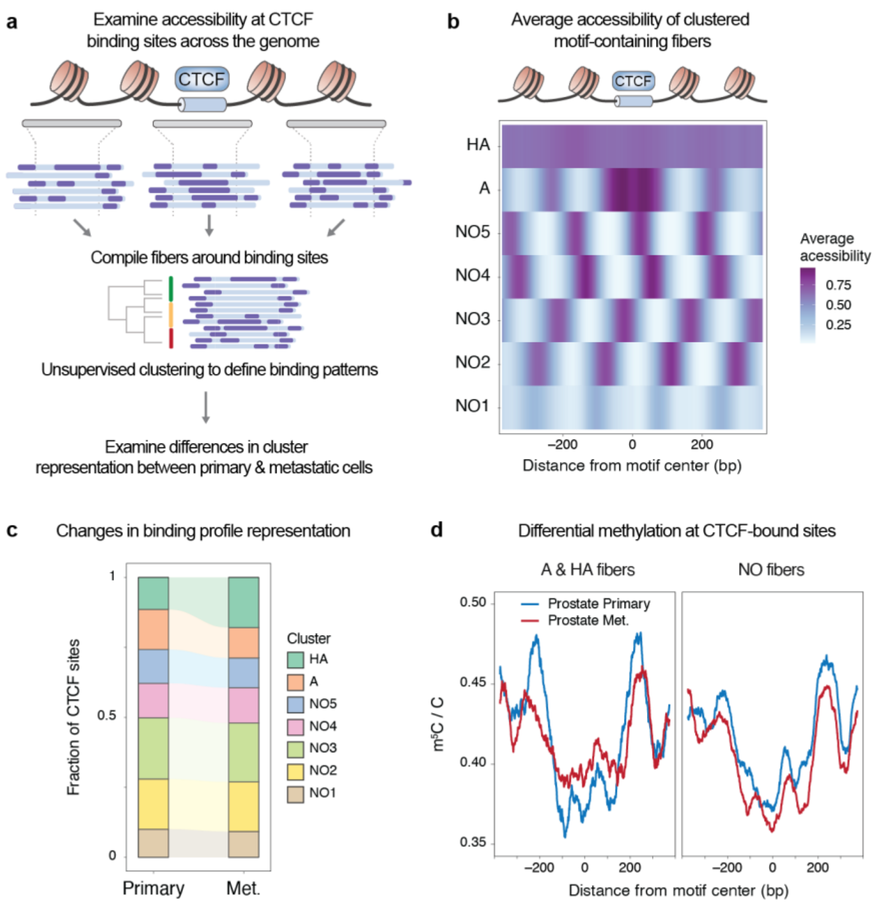


**Supplementary Figure 17: Differential single-molecule chromatin accessibility at CTCF sites in primary and metastatic prostate cancer PDXs. (a)** Overview of framework for analyzing CTCF motif accessibility on individual chromatin fibers from SAMOSA-Tag of primary and metastatic prostate tumour PDXs. **(b)** Unsupervised *Leiden* clustering of single-molecule chromatin accessibility centered at CTCF motifs identified 7 different occupancy states (differentially colored): 5 nucleosome occupied (NO) states with varying nucleosomal registers around the CTCF motif (NO1 – NO5), and 2 accessible states termed ‘A’ (with characteristically phased nucleosomes flanking occupied CTCF motifs) and ‘HA’ (hyper-accessibility to EcoGII within the entire 750-nt window). **(c)** Alluvial plot of shifts in occupancy state distribution between primary tumour and metastasis with notable increase in cluster HA and decrease in cluster A in metastatic cells. **(d)** Co-measurement of $m^6dA$ accessibility and CpG methylation in fibers of type A and HA (left) and NO (right). In metastatic cells compared to primary tumor, while accessible / hyper-accessible CTCF motifs are slightly hypermethylated, CTCF sites in the NO state have this effect reversed with subtle hypomethylation.

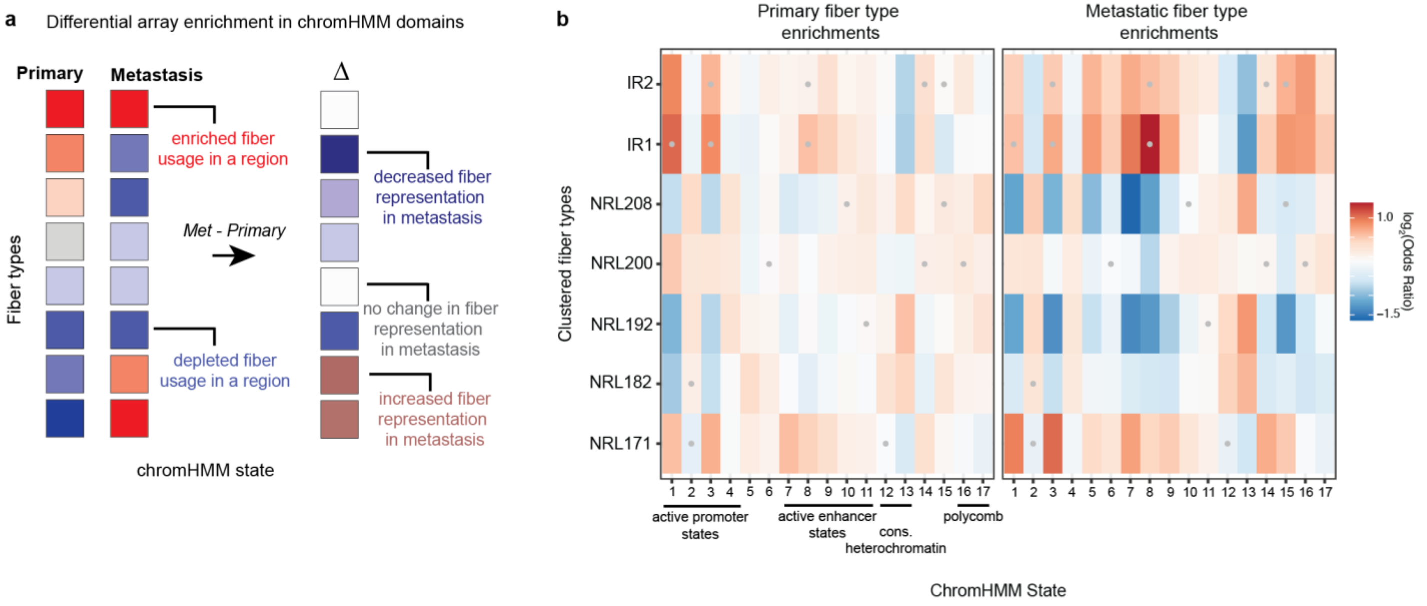


**Supplementary Figure 18: Differential and per-sample fiber-type enrichments in primary and metastatic PDXs. (a)** Overview of the approach for computing a statistic "delta" (Δ) which aims to quantify differential representation of fiber types in specific chromHMM domains across the human epigenome. Beginning with per-domain enrichments in each sample and associated counts, we compute an estimated effect-size (Δ) and associated Storey's $q$ values to correct for multiple testing using a logistic regression analysis and visualize these data in heatmap form with different color scales. **(b)** One-sided Fisher's exact test results for each sample (primary vs. met) for clustered fiber types (signal averages shown in **Fig. 5b**). Red indicates an over-representation of that fiber type (y-axis) within the domain (x-axis); blue indicates a depletion of a fiber type within a domain. Grey dots designate tests that are not significant (N.S., $q > 0.05$). Chromatin state legends: 1: TSS, 2: TSS Flank, 3: TSS Flank Upstream, 4:TSS Flank Downstream, 5: Transcribed region, 6: Weakly transcribed region, 7: Genic enhancer 1, 8: Genic enhancer 2, 9: Active enhancer 1, 10: Active enhancer 2, 11: Weak enhancer, 12: KRAB zinc finger / repetitive region, 13: Constitutive heterochromatin, 14: Bivalently-marked TSS, 15: Bivalently-marked enhancer, 16: Polycomb repressed, 17: Weakly polycomb repressed.

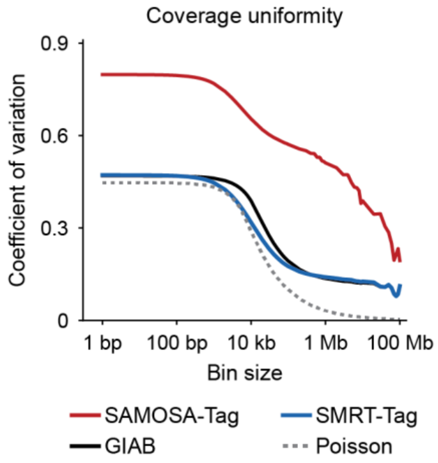


**Supplementary Figure 19: Coverage uniformity of tagmentation- and ligation-based libraries.** Rarefaction curves demonstrating differences in coverage uniformity at varying window sizes across the genome for SAMOSA-Tag (red), SMRT-Tag (blue), ligation-based GIAB PacBio data (black) compared against a random control based on Poisson sampling of reads from the human genome (dashed).
