# Supplementary material for: Direct transposition of native DNA for sensitive multimodal single-molecule sequencing
Source: Nat Genet. 2024 May 9;56(6):1300–9. doi: 10.1038/s41588-024-01748-0 (PMC11176058; doi:10.1038/s41588-024-01748-0)
Supplement: Supplementary file 2 — Reporting Summary [file 41588_2024_1748_MOESM2_ESM.pdf]

nature portfolio

Corresponding author(s): Vijay Ramani, Sivakanthan Kasinathan

Last updated by author(s): Mar 10, 2024

Reporting Summary

Nature Portfolio wishes to improve the reproducibility of the work that we publish. This form provides structure for consistency and transparency in reporting. For further information on Nature Portfolio policies, see our Editorial Policies and the Editorial Policy Checklist.

Statistics

For all statistical analyses, confirm that the following items are present in the figure legend, table legend, main text, or Methods section.

n/a	Confirmed	
☐	☒	The exact sample size (*n*) for each experimental group/condition, given as a discrete number and unit of measurement
☐	☒	A statement on whether measurements were taken from distinct samples or whether the same sample was measured repeatedly
☐	☒	The statistical test(s) used AND whether they are one- or two-sided<br>*Only common tests should be described solely by name; describe more complex techniques in the Methods section.*
☐	☒	A description of all covariates tested
☐	☒	A description of any assumptions or corrections, such as tests of normality and adjustment for multiple comparisons
☐	☒	A full description of the statistical parameters including central tendency (e.g. means) or other basic estimates (e.g. regression coefficient) AND variation (e.g. standard deviation) or associated estimates of uncertainty (e.g. confidence intervals)
☐	☒	For null hypothesis testing, the test statistic (e.g. *F*, *t*, *r*) with confidence intervals, effect sizes, degrees of freedom and *P* value noted<br>*Give P values as exact values whenever suitable.*
☒	☐	For Bayesian analysis, information on the choice of priors and Markov chain Monte Carlo settings
☒	☐	For hierarchical and complex designs, identification of the appropriate level for tests and full reporting of outcomes
☐	☒	Estimates of effect sizes (e.g. Cohen's *d*, Pearson's *r*), indicating how they were calculated

Our web collection on statistics for biologists contains articles on many of the points above.

Software and code

Policy information about availability of computer code

Data collection	Sequencing data was collected using a Pacific Biosciences Sequel II running SMRTlink 11.0.0.146107. Raw data was processed using ccs (Pacific Biosciences, v6.4.0) and demultiplexed using lima (Pacific Biosciences, v2.6.0). Data from libraries sequenced on multiple flow cells was merged using pbmerge (v1.0.0). Images of agarose electrophoresis gels were collected using an Odyssey XF imaging system (LI-COR, software v1.1.0.61). FACS data were collected on a FACS Aria II with FACS Diva v9.0.1 (BD Biosciences).
Data analysis	All scripts required to perform the analyses described in this study and a complete list of required software are available via GitHub at https://github.com/RamaniLab/SMRT-Tag. Scripts utilize Python (v3.8.8) and R (v4.2.1), as well as the following packages and tools: samtools (v1.15.1), bcftools (v1.15.1), bedtools (v2.30.0), mosdepth (v0.3.3), ccs (v6.4.0), pbmm2 (v1.9.0) with minimap2 (v2.15), lima (v2.6.0), primrose (v1.3.0), hap.py (v0.3.12), Truvari (v3.3.0), deepvariant (v1.4.0), pbsv (v2.8.0). FACS data were processed and visualized with FlowJo (v10.8.2, BD Biosciences). SAMOSA-Tag fibers were visualized using a modified version of IGV v2.17.3, available at https://github.com/RamaniLab/SMRT-Tag/tree/main/igv-vis.

For manuscripts utilizing custom algorithms or software that are central to the research but not yet described in published literature, software must be made available to editors and reviewers. We strongly encourage code deposition in a community repository (e.g. GitHub). See the Nature Portfolio guidelines for submitting code & software for further information.

Data

Policy information about availability of data

All manuscripts must include a data availability statement. This statement should provide the following information, where applicable:

- Accession codes, unique identifiers, or web links for publicly available datasets
- A description of any restrictions on data availability
- For clinical datasets or third party data, please ensure that the statement adheres to our policy

SMRT-Tag data are deposited in the NCBI Sequence Read Archive (SRA; accession number PRJNA863422). OS152 and mESC SAMOSA-Tag data, including subreads and kinetic parameters, are deposited in the Gene Expression Omnibus (GEO; accession number GSE225314). PDX SAMOSA-Tag data are available via the controlled access dbGaP repository (study accession phs003511.v1). The following reference genome assemblies or annotations were used in this study: hs37d5 GRCh37, hg38, GRCm38, a concatenated hg38/GRCm39 reference, GENCODE V28 and M25, and UCSC hg19 tandem repeats. NIST/GIAB GRCh37 genome stratifications (v3.0), small variant benchmarks for HG002, HG003, and HG004 (v4.2.1), and Tier 1 SV calls for HG002 (v0.6) were obtained from NCBI (see below). The following additional publicly accessible datasets were used: GIAB-generated PacBio Sequel II HiFi reads from HG002 (SRA accession SRX5527202), Bismark CpG methylation calls from bisulfite sequencing of HG002 (ONT Benchmark Datasets; see below), CTCF binding sites determined by ChIP-seq in U2OS (GEO accession GSE87831) and LNCaP (ENCODE accession ENCFF275GDH) cells, and chromHMM annotations for normal prostate (NGDC accession OMIX237-64-02; https://ngdc.cncb.ac.cn/omix/release/OMIX237).

hg19 tandem repeats in BED format were downloaded from UCSC:
ftp://hgdownload.soe.ucsc.edu/goldenPath/hg19/bigZips/hg19.trf.bed.gz

VCF and BED files for NIST/GIAB small variant benchmarks were obtained from:
ftp://ftp-trace.ncbi.nlm.nih.gov/giab/ftp/release/AshkenazimTrio/HG002_NA24385_son/NISTv4.2.1/GRCh37/
ftp://ftp-trace.ncbi.nlm.nih.gov/giab/ftp/release/AshkenazimTrio/HG003_NA24149_father/NISTv4.2.1/GRCh37/
ftp://ftp-trace.ncbi.nlm.nih.gov/giab/ftp/release/AshkenazimTrio/HG004_NA24143_mother/NISTv4.2.1/GRCh37/

GIAB genome stratifications were downloaded from:
ftp://ftp-trace.ncbi.nlm.nih.gov/giab/ftp/release/genome-stratifications/v3.0/v3.0-stratifications-GRCh37.tar.gz

GIAB-generated HG002 PacBio Sequel II HiFi data were downloaded from:
ftp://ftp-trace.ncbi.nlm.nih.gov/giab/ftp/data/AshkenazimTrio/HG002_NA24385_son/PacBio_SequelII_CCS_11kb/
HG002.SequelII.pbmm2.hs37d5.whatshap.haplotag.RTG.10x.trio.bam

GIAB Tier 1 SV calls for HG002 were downloaded from:
https://ftp-trace.ncbi.nlm.nih.gov/ReferenceSamples/giab/release/AshkenazimTrio/HG002_NA24385_son/NIST_SV_v0.6/HG002_SVs_Tier1_v0.6.bed
https://ftp-trace.ncbi.nlm.nih.gov/ReferenceSamples/giab/release/AshkenazimTrio/HG002_NA24385_son/NIST_SV_v0.6/HG002_SVs_Tier1_v0.6.vcf.gz

HG002 bisulfite sequencing CpG methylation calls were downloaded from:
https://ont-open-data.s3.amazonaws.com/gm24385_mod_2021.09/bisulphite/cpg/CpG.gz.bismark.zero.cov.gz

Research involving human participants, their data, or biological material

Policy information about studies with human participants or human data. See also policy information about sex, gender (identity/presentation), and sexual orientation and race, ethnicity and racism.

Reporting on sex and gender	Tissues used to derive prostate cancer xenografts were donated by one participant of male sex. Sex and gender were not considered in study design.
Reporting on race, ethnicity, or other socially relevant groupings	No socially relevant categorization variables were reported or used in the study.
Population characteristics	Tissues used to derive prostate cancer xenografts were donated by a 71-year-old male participant with metastatic castration-resistant prostate cancer.
Recruitment	The participant who donated tissue for derivation of prostate cancer xenografts was recruited from the UCSF Urology service.
Ethics oversight	This study is covered by an active human subjects approval (protocol number 90911 'Use of Marker in Cytometric Analysis in Prostate Cancer to predict biological potential' UCSF IRB 11-05226).

Note that full information on the approval of the study protocol must also be provided in the manuscript.

Field-specific reporting

Please select the one below that is the best fit for your research. If you are not sure, read the appropriate sections before making your selection.

☒ Life sciences ☐ Behavioural & social sciences ☐ Ecological, evolutionary & environmental sciences

For a reference copy of the document with all sections, see nature.com/documents/nr-reporting-summary-flat.pdf

Life sciences study design

All studies must disclose on these points even when the disclosure is negative.

Sample size	No specific calculation was performed to establish a required sample size for experiments in this study.<br><br>In experiments where specific, relevant conditions were tested that eventually were incorporated into the final SMRT-Tag method (i.e gap repair optimization, top hits) a minimum of two technical replicates were generated as a trade off between reagent usage and accuracy in estimating reaction efficiency.<br><br>The number of replicate SAMOSA-Tag experiments was determined to be a minimum of 3 based on previous experiments analyzing m6dA footprinting data (see Abdulhay et al. 2019, eLife, doi: 10.7554/eLife.59404) and was increased to n=8 for our OS152 and mESC SAMOSA-Tag experiments and n=6 for both primary and metastasis PDX SAMOSA-Tag experiments, to improve our analysis of reproducibility.
Data exclusions	For both OS152 SAMOSA-Tag and PDX SAMOSA-Tag analyses, unmethylated or lowly m6dA methylated fibers were excluded on the basis of lack of m6dA signal for determining nucleosome footprints. For OS152 SAMOSA-Tag data, across all n=8 replicates, these excluded fibers accounted for ~12.5% of all examined fibers. For PDX SAMOSA-Tag data (both primary and met.) across all n=6 replicates, these excluded fibers accounted for ~1.5 % of all examined fibers.
Replication	To verify reproducibility for SMRT-Tag gap repair optimization, the top performing gap-repair conditions were tested on multiple input samples, as well as with different amounts of input DNA. For each test, repair efficiency was ascertained by yield as well as fragment size distribution – see Supplementary Figures 1. and 2, and Supplementary Table 2 and Table 3. Selected repair conditions (Phu/Taq) performed adequately across a range of inputs, and library preparation yields were generally consistent when stratified by input amount and type.<br><br>For OS152 SAMOSA-Tag experiments, n=8 replicates were generated, and downstream analyses determining fiber type distributions and enrichments performed independently to validate results were reproducible. Supplementary Figure 14 compares fiber type enrichment patterns (odds ratios) across technical replicates, and demonstrates 1) that fiber types discovered via SAMOSA-Tag are reproducible across replicates, and 2) that all 8 replicates are highly consistent with each other. The same analysis performed separately on primary and met. PDX SAMOSA-Tag datasets, n=6 replicates, also indicated a high level of consistency.
Randomization	We did not perform any experiments that required randomization. Our study focused primarily on developing a new method, and demonstrating its utility by profiling relevant samples.
Blinding	We did not perform any experiments that required explicit blinding. For fiber type and binding site cluster determination, clustering analyses on single molecule fibers were performed using an unsupervised (leiden) clustering algorithm. Blinding is not required or routinely performed for this genomic analysis.

Reporting for specific materials, systems and methods

We require information from authors about some types of materials, experimental systems and methods used in many studies. Here, indicate whether each material, system or method listed is relevant to your study. If you are not sure if a list item applies to your research, read the appropriate section before selecting a response.

Materials & experimental systems

n/a	Involved in the study	
☐	☒	Antibodies
☐	☒	Eukaryotic cell lines
☒	☐	Palaeontology and archaeology
☐	☒	Animals and other organisms
☒	☐	Clinical data
☒	☐	Dual use research of concern
☒	☐	Plants

Methods

n/a	Involved in the study	
☒	☐	ChIP-seq
☐	☒	Flow cytometry
☒	☐	MRI-based neuroimaging

Antibodies

Antibodies used	PE anti-mouse H-2 antibody (BioLegend 125505) was used at dilution 1 µg antibody per 8 – 12.5 x 10^6 cells
Validation	Per the manufacturer, "Each lot of this antibody is quality control tested by immunofluorescent staining with flow cytometric analysis." Also per the manufacturer, relevant citations include:<br>Boyd DF, et al. 2020. Nature. 587:466.<br>Pyzik M, et al. 2014. J Immunol. 193:6061.<br>Oyarce C, et al. 2018. Front Immunol. 8:1794.<br>Bockerstett KA, et al. 2018. Int J Mol Sci. 19:E1096.<br>Saunderson S and McLellan A. 2017. J Immunol. 10.4049/jimmunol.1601537.

Eukaryotic cell lines

Policy information about cell lines and Sex and Gender in Research

Cell line source(s)	Metastatic osteosarcoma cell line OS152 was provided by Alejandro Sweet-Cordero at the University of California, San Francisco. Mouse embryonic stem cells were a gift from Elphege Nora at the University of California, San Francisco.
Authentication	The authenticity of the OS152 cell line was confirmed by genotyping following a protocol previously used (see Sayles et al. 2019, Cancer Discovery, doi: 10.1158/2159-8290.CD-17-1152) using CellCheck 9 Plus (IDEXX BioAnalytics). mESC E14 cells were not authenticated directly, though genotyping data from PacBio sequencing of cells from this line used in various studies appear concordant.
Mycoplasma contamination	The OS152 cell line was tested for mycoplasma contamination, and aliquots used in this study were confirmed to be negative for mycoplasma. The mESC E14 cell line used in this study was also tested for mycoplasma contamination and confirmed to be negative for mycoplasma.
Commonly misidentified lines (See ICLAC register)	No commonly misidentified cell lines were used in this study.

Animals and other research organisms

Policy information about studies involving animals; ARRIVE guidelines recommended for reporting animal research, and Sex and Gender in Research

Laboratory animals	Patient-derived xenografts were implanted subcutaneously into 6- to 8-week-old male NOD scid gamma (NSG) mice (UCSF Breeding Core) maintained under specific pathogen-free conditions.
Wild animals	n/a
Reporting on sex	Sex was not considered in the study design.
Field-collected samples	The study did not involve field-collected samples.
Ethics oversight	Experiments were performed under a protocol approved by the UCSF Institutional Animal Care and Use Committee (IACUC; number AN195508).

Note that full information on the approval of the study protocol must also be provided in the manuscript.

Plants

Seed stocks	n/a
Novel plant genotypes	n/a
Authentication	n/a

Flow Cytometry

Plots

Confirm that:

- [x] The axis labels state the marker and fluorochrome used (e.g. CD4-FITC).
- [x] The axis scales are clearly visible. Include numbers along axes only for bottom left plot of group (a 'group' is an analysis of identical markers).
- [x] All plots are contour plots with outliers or pseudocolor plots.
- [x] A numerical value for number of cells or percentage (with statistics) is provided.

Methodology

Sample preparation	Primary and metastasis PDX models derived from one patient with both primary and lymph-metastatic prostate cancer were used in this study. Creation of these PDX models is described in (Nguyen et al, 2018, Science Translational Medicine). PDX tumors were passaged in NSG mice, with verification of tumor similarity to the original biopsy via histopathological and

growth-rate based comparisons. On the day of processing, samples were first surgically removed from mice, placed into sterile collection buffer, and dissociated manually using sterile surgical blades, as well as using enzymatic treatment (see Methods). Resulting single cell suspensions were washed via centrifugation at 4ºC with PBS, strained to remove aggregates through a 70µm filter, and then stained in Cell Staining Buffer (Biolegend) with 1µg PE anti-mouse H-2 Antibody (Biolegend, Cat# 125505), and 1µL of SYTOX Red Dead Cell Stain (Thermo Fisher).

Instrument

BD FACS ARIA II (BD Biosciences)

Software

Data was acquired with FACS DIVA (v9.0.1, BD Biosciences) and was visualized and analyzed using FlowJo (v.10.8.2, BD Biosciences)

Cell population abundance

The relevant cell population (not mouse, not dead) was determined to be ~ 16.05% of the primary PDX and ~ 14.3% for the metastasis PDX sample via FACS. SAMOSA-Tag libraries prepared from the resulting population were mapped to both human and mouse genomes, and sample purity as estimated by the fraction of human alignments is between 27.5 - 32.1% for the primary PDX and 96.3-96.7% for the metastasis PDX.

Gating strategy

Cell singlets were selected by gating on forward scatter. Subsequently, an APC negative gate corresponding to live cells (SYTOX Red) was defined by calibrating against a single-stain control. A PE negative gate corresponding to "not-mouse" cells was similarly defined by calibrating against a single-stain control. In both cases, the gate was set at the minimum between the negative and positive signal peaks in the single-stain controls. The intersection of the two gates defined the relevant cell population. Supplementary Figure 15 exemplifies the gating strategy.

☒ Tick this box to confirm that a figure exemplifying the gating strategy is provided in the Supplementary Information.
