# Supplementary material for: Direct transposition of native DNA for sensitive multimodal single-molecule sequencing
Source: Nat Genet. 2024 May 9;56(6):1300–9. doi: 10.1038/s41588-024-01748-0 (PMC11176058; doi:10.1038/s41588-024-01748-0)
Supplement: Supplementary file 8 — Unprocessed analytical agarose gel for Fig. 1b. [file 41588_2024_1748_MOESM8_ESM.pdf]

Source Data for Fig. 1b

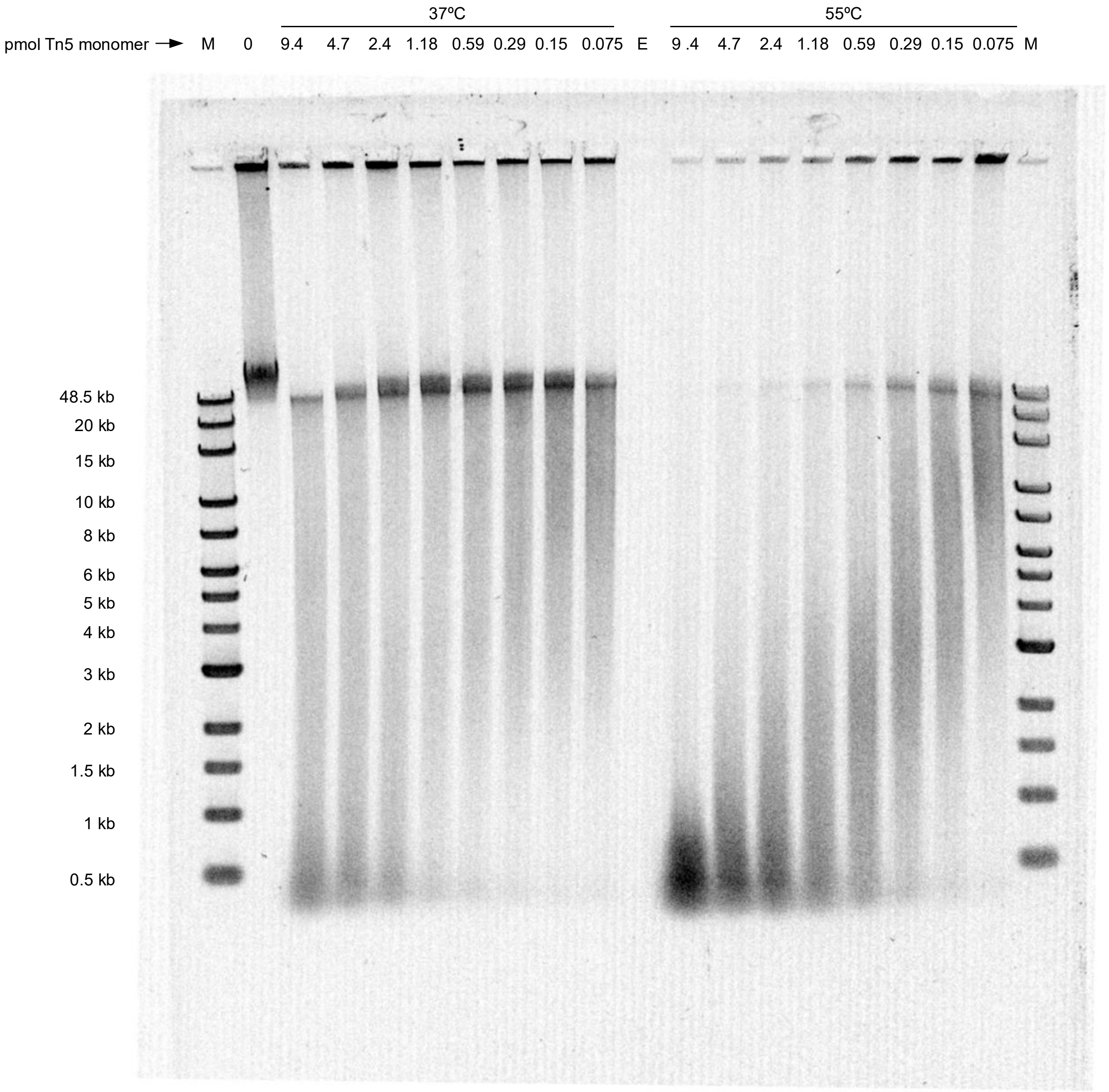

Tagmentation duration: 30 minutes.

M = Marker, E = Empty.
0.5% (w/v) TAE/agarose gel stained with SYBR gold and imaged on LI-COR Odyssey FX scanner.
